# Perilipin 5 deletion protects against nonalcoholic fatty liver disease and hepatocellular carcinoma by modulating lipid metabolism and inflammatory responses

**DOI:** 10.1038/s41420-024-01860-4

**Published:** 2024-02-22

**Authors:** Paola Berenice Mass-Sanchez, Marinela Krizanac, Paula Štancl, Marvin Leopold, Kathrin M. Engel, Eva Miriam Buhl, Josef van Helden, Nikolaus Gassler, Jürgen Schiller, Rosa Karlić, Diana Möckel, Twan Lammers, Steffen K. Meurer, Ralf Weiskirchen, Anastasia Asimakopoulos

**Affiliations:** 1 Institute of Molecular Pathobiochemistry, Experimental Gene Therapy and Clinical Chemistry (IFMPEGKC), RWTH University Hospital Aachen, D-52074 Aachen, Germany; 2 Bioinformatics Group, Division of Molecular Biology, Department of Biology, Faculty of Science, University of Zagreb, HR-10000 Zagreb, Croatia; 3 Institute for Medical Physics and Biophysics, Leipzig University, Facutly of Medicine, D-04107 Leipzig, Germany; 4 Electron Microscopy Facility, Institute of Pathology, RWTH Aachen University Hospital, D-52074 Aachen, Germany; 5 MLM Medical Labs, D-41066 Mönchengladbach, Germany; 6 Section Pathology, Institute of Legal Medicine, University Hospital Jena, D-07747 Jena, Germany; 7 Institute for Experimental Molecular Imaging, RWTH Aachen, D-52074 Aachen, Germany

**Keywords:** Gastrointestinal diseases, Cell biology

## Abstract

The molecular mechanisms underlying the transition from nonalcoholic fatty liver disease (NAFLD) to hepatocellular carcinoma (HCC) are incompletely understood. During the development of NAFLD, Perilipin 5 (PLIN5) can regulate lipid metabolism by suppressing lipolysis and preventing lipotoxicity. Other reports suggest that the lack of PLIN5 decreases hepatic injury, indicating a protective role in NAFLD pathology. To better understand the role of PLIN5 in liver disease, we established mouse models of NAFLD and NAFLD-induced HCC, in which wild-type and *Plin5* null mice were exposed to a single dose of acetone or 7,12-dimethylbenz[a]anthracene (DMBA) in acetone, followed by a 30-week high-fat diet supplemented with glucose/fructose. In the NAFLD model, RNA-seq revealed significant changes in genes related to lipid metabolism and immune response. At the intermediate level, pathways such as AMP-activated protein kinase (AMPK), signal transducer and activator of transcription 3 (STAT3), c-Jun N-terminal kinase (JNK), and protein kinase B (AKT) were blunted in *Plin5-*deficient mice (*Plin5*^−*/*−^) compared to wild-type mice (WT). In the NAFLD-HCC model, only WT mice developed liver tumors, while *Plin5*^*−/−*^ mice were resistant to tumorigenesis. Furthermore, only 32 differentially expressed genes associated with NALFD progession were identified in *Plin5* null mice. The markers of mitochondrial function and immune response, such as the peroxisome proliferator‐activated receptor-γ, coactivator 1‐α (PGC-1α) and phosphorylated STAT3, were decreased. Lipidomic analysis revealed differential levels of some sphingomyelins between WT and *Plin5*^*−/−*^ mice. Interestingly, these changes were not detected in the HCC model, indicating a possible shift in the metabolism of sphingomelins during carcinogenesis.

## Introduction

Nonalcoholic fatty liver disease (NAFLD) is one of the leading causes of liver disease worldwide, with an estimated global incidence of 47 cases per 1000 person-years [1]. NAFLD has been described as a spectrum of chronic liver diseases marked by an accumulation of liver lipids and an excess of triacylglycerols (TAG) [2]. Inflammation and the resulting liver injury promote the switch from NAFLD to nonalcoholic steatohepatitis (NASH). Untreated NASH can lead to the development of liver fibrosis, cirrhosis, and ultimately to HCC [2–4]. Noticeably, liver cancer is one of the top three causes of cancer deaths in 46 countries [5]. Although the incidence of NAFLD-induced HCC (NAFLD-HCC) is lower than HCC caused by other etiologies, NAFLD is more prevalent than other liver diseases [6]. However, the molecular mechanisms explaining the transition from NAFLD to NAFLD-HCC remain poorly understood.

The members of the perilipin protein family play an important role in lipid metabolism and the pathogenesis of NAFLD. Of particular interest is Perilipin 5 (PLIN5), which is anchored to the surface of the lipid droplet (LD), thus allowing its stabilization and promoting LD-mitochondria contacts [7]. PLIN5 affects lipolysis by competitive binding to comparative gene identification 58 (CGI-58) and adipose triglyceride lipase (ATGL) [7, 8]. Furthermore, it acts as a coregulator of the receptor- coactivator 1 activated by peroxisome proliferator‐activated receptor-γ coactivator 1‐α (PGC-1α), regulating oxidative capacity and mitochondrial efficiency [9].

Different models of NAFLD have been developed to study *Plin5* functions [10–13]. However, information on its role in NAFLD-HCC is limited. PLIN5 expression is increased in human HCC livers with exacerbated inflammation but not in non-HCC livers [14]. Finally, specific splice variants such as PLIN5 46808AT are more common in primary sites of HCC with metastasis than in primary HCC [15].

Because its functions are not limited only to the LD coating, but also include lipolysis, oxidative stress, stress of the endoplasmic reticulum, inflammation, and autophagy, *Plin5* presents itself as a noteworthy target for investigation [10, 16–19]. Therefore, PLIN5 may have important biological functions in the progression from NAFLD to HCC. To further elucidate the role of *Plin5* during the progression to HCC, we performed studies in a NAFLD and a NAFLD-HCC model. We demonstrate that the lack of *Plin5* prevents liver injury and inhibits liver tumorigenesis by regulating key signaling pathways associated with carcinogenesis.

## Results

### *Plin5* deletion reduces steatosis in NAFLD and hepatic tumor development in NAFLD-HCC

**Fig. 1:**
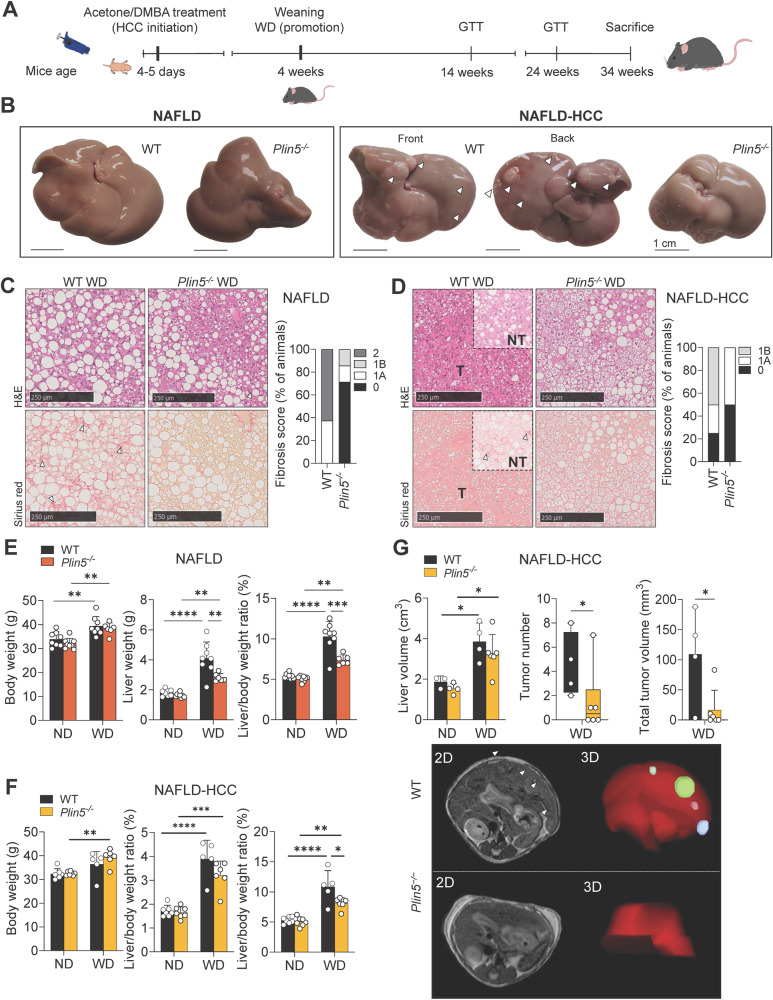
Perilipin 5 knockout reduces fibrosis in a NAFLD model and inhibits hepatic tumorigenesis in a NAFLD-HCC model. **A** Schematic diagram of the study design. To promote the development of NAFLD and hepatic tumors, mice were administered with a combination of a single application of either acetone (NAFLD model) or DMBA dissolved in acetone (NAFLD-HCC model) at p4-5. At the end of the weaning period, mice were fed with either a ND or WD for 30 weeks before sacrifice. At weeks 14 and 24 of the experiment, glucose tolerance test was performed. Mice were sacrificed 30 weeks after the initiation of the diet. **B** Representative macroscopic appearance of the WT and *Plin5*^*−/−*^ livers corresponding to the NAFLD and NAFLD-HCC models. WT mice of the NAFLD-HCC model exhibited hepatic tumors indicated by white arrowheads. **C**, **D** Representative histopathology of the hepatic tissue and fibrosis scoring of mice subjected to the **C** NAFLD and **D** NAFLD-HCC (NT, non-tumor; T, tumor) model. Upper panels show H&E and lower panels show Sirius red staining. Collagen deposits are marked by white arrowheads. **E**, **F** Body weight, liver weight, and liver/body weight of the **E** NAFLD model (n = 6–8 mice per group) and the **F** NAFLD-HCC model (5–7 mice per group). **G** Analysis of the CT scans showing liver volume, tumor number and total tumor volume of mice used in the NAFLD-HCC model. 2D- and 3D-rendering images in the lower panel are representative results obtained from WT and *Plin5*^*−/−*^ mice. Tumors observed in the 3D rendering are marked by arrowheads. Data represent mean ± SD and were analyzed by two-way ANOVA using Tukey’s post-test. * *p* < 0.05, ** *p* < 0.01, *** *p* < 0.001, **** *p* < 0.0001.

To better understand *Plin5* functions in the context of NAFLD and its progression to HCC, we used a modified version of a model previously described [20] (Fig. 1A). The experimental setup resulted in two groups: (i) animals treated with acetone and fed a Western diet (WD) representing NAFLD, and (ii) animals treated with DMBA and fed a WD representing the NAFLD-HCC model. We first confirmed the up-regulation of PLIN5 in both models (Suppl. Fig. 1A). Macroscopic examination of the livers showed that all mice fed WD exhibited hepatomegaly. Furthermore, only wild-type (WT) mice treated with DMBA and WD developed liver tumors, while *Plin5*^*−/−*^ mice were refractory to tumor development (Fig. 1B). Histological analysis of WD-fed mice revealed morphological changes in hepatocytes and the presence of steatosis in both models. In the NAFLD model, all WT mice developed distinctive fibrosis, while only 30% of *Plin5*^*−/−*^ mice developed low fibrosis scores (Fig. 1C). In the NAFLD-HCC model, the tumor area in the livers of WT mice was characterized by mixed vacuolated hepatocytes, while *Plin5*^-/-^ mice only showed lipid accumulation (Fig. 1D). When analyzing fibrosis development in the NAFLD-HCC model, we found that 75% of the WT mice fed with WD showed low scores of fibrosis, while 50% of *Plin5*^*−/−*^ mice under the same treatment did not develop fibrosis at all.

After 30 weeks of diet, the mice of the NAFLD model presented a similar body weight gain independent of the genotype (Fig. 1E). However, liver weights (*p* < 0.01, two-way ANOVA using Tukey’s post-test) and values for liver/body weight ratios (*p* < 0.001, two-way ANOVA using Tukey’s post-test) were significantly reduced in *Plin5*^−/−^ mice fed WD compared to WT littermates. Computed tomography (CT) revealed accumulation of visceral and subcutaneous fat in animals fed with WD (Suppl. Figure1 B-E). Furthermore, *Plin5*^−/−^ mice presented a lower ratio of body fat volume/body volume compared to WT when fed with control diet (Suppl. Figure 1D).

In the NAFLD-HCC model, only the liver/body weight ratio was significantly reduced in *Plin5*^−/−^ mice fed with WD (*p* < 0.05, two-way ANOVA using Tukey’s post-test) (Fig. 1F). CT scans confirmed the higher presence of tumors in WT (Fig. 1G), while most *Plin5*^*−/−*^ mice developed no tumors. However, the ones developed had smaller tumors than those found in WT, showing that *Plin5* deficiency protects against hepatic tumor formation.

### *Plin5* deletion prevents liver injury in NAFLD

**Fig. 2:**
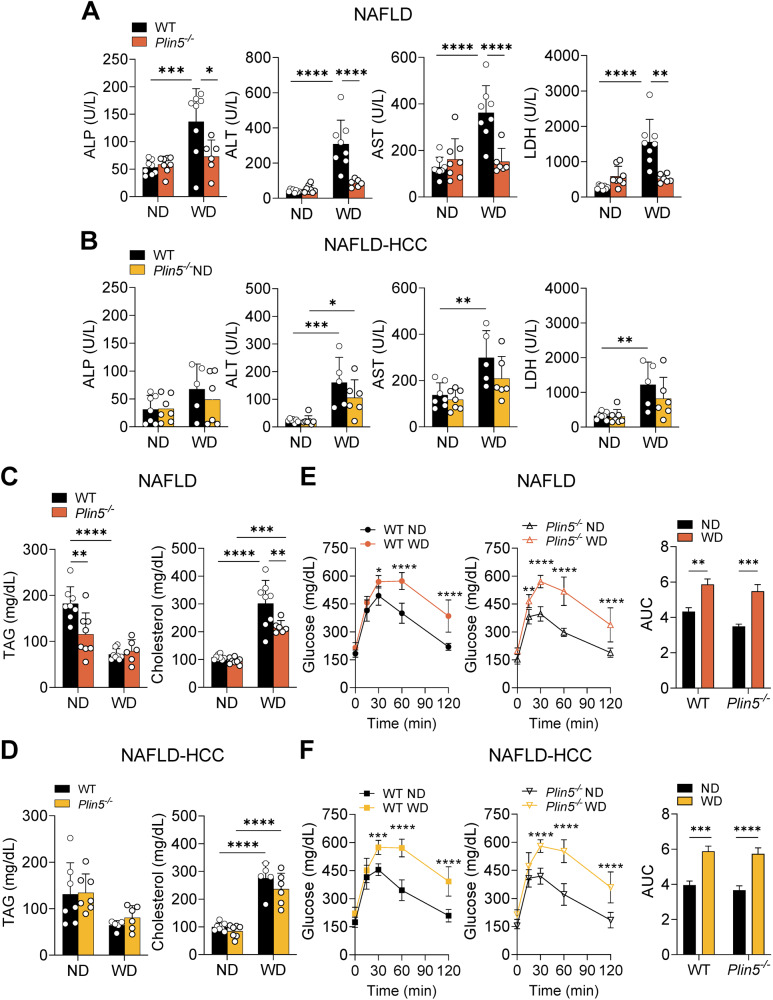
Depletion of *Plin5* ameliorates hepatic liver injury in NAFLD. **A**, **B** Alkaline phosphatase (ALP), alanine transaminase (ALT), aspartate transaminase (AST) and lactate dehydrogenase (LDH) in serum of mice of the **A** NAFLD and **B** NAFLD-HCC groups (*n* = 5–8 mice per group). **C**, **D** Serum triacylglycerol (TAG) and cholesterol levels in mice of the **C** NAFLD and **D** NAFLD-HCC models (*n* = 5–8 mice per group). **E**, **F** Glucose tolerance test 24 weeks (20 weeks after beginning of the diet) after the beginning of the experiment of the **E** NAFLD and **F** NAFLD-HCC models (*n* = 5–8 mice per group). Data represent mean ± SD except in calculation of AUC where data are represented as mean ± SE. All data were analyzed by two-way ANOVA using Tukey’s post-test. *p < 0.05, **p < 0.01, ***p < 0.001, ****p < 0.0001.

We previously reported that *Plin5* depletion prevents liver injury in mice exposed to a high-fat diet (HFD) [11]. Similarly, in the NALFD model, serum levels of liver injury markers including alkaline phosphatase (*p* < 0.05), alanine transaminase (*p* < 0.0001), aspartate transaminase (*p* < 0.0001) and lactate dehydrogenase (*p* < 0.01, all two-way ANOVA using Tukey’s post-test) decreased significantly in *Plin5*^−/−^ mice compared to WT littermates (Fig. 2A). In the NAFLD-HCC model, no significant differences were observed between WT and *Plin5*^−/−^ mice (Fig. 2B). Furthermore, TAG analysis did not reveal significant differences between the *Plin5*^*−/−*^ and WT groups in both models when fed WD, while cholesterol levels of *Plin5*^−/−^ mice fed WD were reduced in the NAFLD model (*p* < 0.01, two-way ANOVA using Tukey’s post-test) (Fig. 2C).

Since HFD consumption results in impaired glucose tolerance [21], we conducted a glucose tolerance test at 10 and 20 weeks in the WD group. This analysis revealed that the WD-fed WT and *Plin5*^−/−^ mice both presented impaired glucose tolerance already after 10 weeks (Suppl. Fig. 2). This trend continued after 20 weeks from diet initiation (Fig. 2E, F).

Together, these results indicate that *Plin5* deficiency decreases hepatic injury in NAFLD, whereas only a subtle decrease is provoked in the NAFLD-HCC model. Furthermore, WD imposed alterations in TAGs and caused impairment in glucose metabolism in both genotypes and both models, while *Plin5*^−/−^ mice fed WD also had lower cholesterol levels in the NAFLD model.

### *Plin5* depletion decreases mitochondrial oxidation in NAFLD but not in NAFLD-HCC

**Fig. 3:**
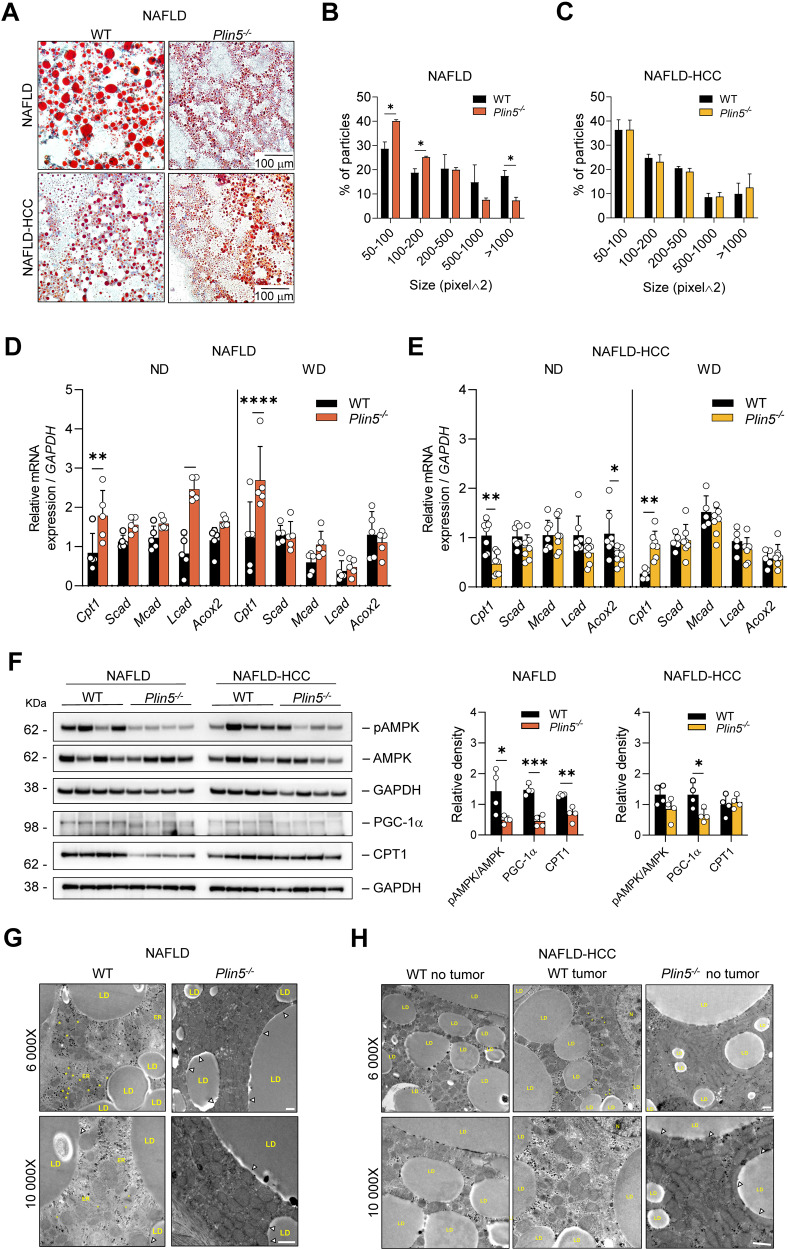
Mitochondrial function is altered in mice lacking *Plin5* in NAFLD and NAFLD-HCC. **A** Representative Oil Red O staining of liver tissue from mice fed with WD representing the NAFLD and NAFLD-HCC model. Images were acquired at 200× magnification (*n* = 2 biological and 2 technical replicates). **B**, **C** Distribution of lipid droplet size from representative HE stains of mice fed with WD in the **B** NAFLD or **C** NAFLD-HCC model (*n* = 3 for each genotype and 5 representative fields per sample). **D**, **E** RT-qPCR determined expression of genes involved in β-oxidation in the liver of mice of the **D** NAFLD and **E** NAFLD-HCC model (*n* = 5 mice per group, two technical replicates per mice). Data represent mean ± SD and were analyzed by two-way ANOVA using Tukey’s post-test. **F** Western blot analysis of whole liver extracts from WT and *Plin5*^*−/−*^ mice of the NAFLD and NAFLD-HCC model. GAPDH was used as a loading control. Images represent the results of at least two independent experiments (left panel). Relative density quantification of Western blots is presented. Measurements were normalized to the loading control GAPDH, except in the case of pAMPK, where measurements were normalized to total levels of AMPK (n = 4 mice per sample) (right panel). In the measurement of relative density of protein expression, *t*-test was performed for each protein. *p < 0.05, **p < 0.01, ***p < 0.001, ****p < 0.0001. **G**, **H** Representative electron microscopy images of hepatic tissue from WT and *Plin5*^*−/−*^ mice fed with a WD from the **G** NAFLD or **D** NAFLD-HCC model (*n* = 3 mice per group). Magnifications are: 6000× and 10,000× as indicated in each panel. Abbreviations/symbols used are: LD lipid droplet, ER endoplasmic reticulum, * peroxisomes; Δ LD-mitochondria contacts. White scales indicate 1000 nm.

To address lipid storage, Oil Red O staining was performed, revealing that the livers of WT and *Plin5*^*−/*−^ mice presented large amounts of intrahepatic lipids after WD feeding (Fig. 3A). Furthermore, analysis of LD size showed that *Plin5*^*−/*−^ mice had an increased number of small LDs and a decreased number of large LDs compared to WT in the NAFLD model (Fig. 3B). On the contrary, DMBA treatment was accompanied by similar LD sizes in WT and *Plin5*^*−/*−^ livers (Fig. 3C).

The catabolism of fatty acids occurs within mitochondria. Consequently, cells favor rapid transfer of fatty acids from LDs to mitochondria [22]. Therefore, we expected changes in markers related to fatty acid oxidation (FAO). RT-qPCR indicated that under normal diet (ND), *Plin5*^*−/−*^ mice showed increased expression of carnitine palmitoyltransferase I (*Cpt1)* and long-chain acyl-CoA dehydrogenase (*Lcad)* (Fig. 3D). Interestingly, when animals were challenged with WD, the differences between *Plin5*^*−/−*^ and WT were blunted and only *Cpt1* levels remained increased in *Plin5*^*−/−*^ mice. In this scenario, *Plin5* depletion seems to increase mitochondrial oxidation only under ND conditions. On the contrary, the results of the NAFLD-HCC model indicated that under WD, *Plin5*^*−/−*^ mice have increased expression of *Cpt1*, while no changes were observed in the rest of the markers analyzed (Fig. 3E). CPT1 protein levels were next evaluated in WT and *Plin5*^*−/−*^ mice exposed to WD. Interestingly, contrary to the transcript level, the CPT1 protein was decreased in *Plin5*^*−/−*^ mice only in the NAFLD model. Moreover, PGC-1α was decreased in *Plin5*^*−/−*^ mice in both models. Interestingly, when we analyzed the common upstream regulator of CPT1 and PGC-1α, AMPK, *Plin5*^*−/−*^ mice tended to present lower levels of phosphorylated AMPK (pAMPK) than control mice, possibly indicating that mitochondrial function is impaired in *Plin5*^*−/−*^ mice fed with WD (Fig. 3F).

Transmission electron microscopy was used to detect potential changes in the appearance and localization of mitochondria within liver tissues. According to previous studies [10, 11], *Plin5*^*−/*−^ mice exhibited increased numbers and enlarged mitochondria, and notably increased physical mitochondrial-LD contacts were observed in *Plin5*^−/−^ mice fed WD (Fig. 3G, H).

These data collectively indicate that mitochondrial impairment is more evident in *Plin5*^*−/−*^ mice in NAFLD than in NAFLD-induced HCC.

### Lipidomic analysis shows that *Plin5* depletion alters serum sphingomyelin content

Previous reports showed an increase in ceramides and sphingomyelins (SM) in the muscle of *Plin5*^*−/−*^ mice [23]. However, the composition of liver lipids under WD and the development of HCC have not been previously reported in the respective mice. Thus, we dissected the lipid profiles by performing a targeted lipidomic analysis.

**Fig. 4:**
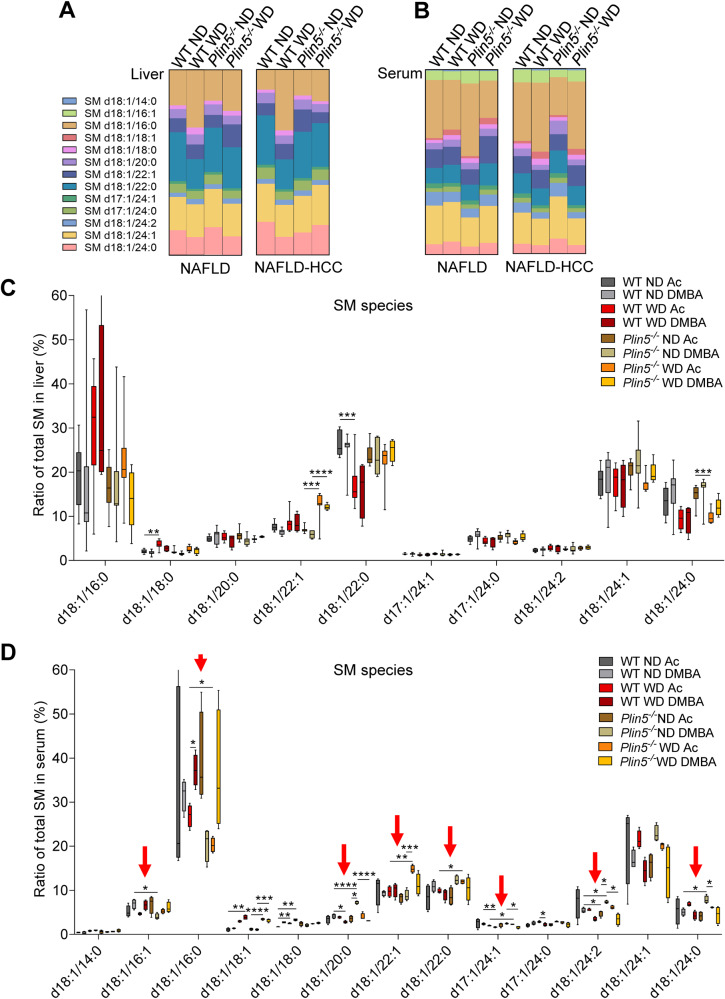
Sphingomyelin profile in serum is altered in *Plin5*^*−/−*^ mice that developed NAFLD. **A**, **B** Visual representation of the individual sphingomyelin (SM) species in (**A**) liver and (**B**) serum samples of WT and *Plin5*^*−/−*^ mice fed with ND or WD from the NAFLD and NAFLD-HCC models. Data are given as % of the total amount of SM. **C** Ratio of total SM in liver presented as percentages (*n* = 4 mice per group). **D** Ratio of total SM in serum presented as percentages (*n* = 4 mice per group). Red arrows indicate differences found between WT and *Plin5*^*−/−*^ mice. For figures (**C**) and (**D**), data are depicted as Box-Whisker-plots representing the smallest and largest value. The line represents the median. Statistical significance was determined using multiple *t* tests and the Holm-Sidak method with *α* = 0.05 for multiple comparison. *p < 0.05, **p < 0.01, ***p < 0.001, ****p < 0.0001.

Therefore, we analyzed the relative fatty acyl compositions of the SM species, phosphatidylinositol (PI), phosphatidylcholine (PC), and phosphatidylethanolamine (PE) fractions in liver and serum samples to assess whether there were differences between individual genotypes and treatments (Suppl. Fig. 3). Interestingly, we found that several species of SM in the liver and serum showed differences between genotypes only in ND. Consistently, the highest differences in individual SM species in the liver (Fig. 4A) and serum (Fig. 4B) were the most prominent in the ND groups. In the liver, no significant differences were found between genotypes were found (Fig. 4C, Suppl. Table 1). In serum, the differences between the treatments and genotypes were more evident in *Plin5*^*−/−*^ mice (Fig. 4B, D and Suppl. Table 1). SM species d18:1/22:1, d18:1/22:0 and d17:1/24:1 were increased, while the main SM d18:1/16:0 was decreased in *Plin5*^*−/−*^ mice in the NAFLD model. These differences were not found in the NAFLD-HCC model.

Differences in total PI abundance in liver and serum were found in *Plin5*^*−/−*^ mice compared to WT mice (cf. Suppl. Fig. 3B). However, when we compared the relative moieties of individual PI species, we found that their abundance in liver and serum was very similar, with PI 38:4 always representing the most abundant species (Suppl. Fig. 4).

Lipidomic analysis revealed that the PC content in serum differed significantly between WT and *Plin5*^−/−^ mice. In particular, increased quantities of PC were noticed in *Plin5*^*−/−*^ mice in the NAFLD model compared with WT mice (cf. Suppl. Fig. 3C). In addition, in serum, *Plin5*^*−/−*^ mice presented model-specific differences in the abundance of PE (cf. Suppl. Fig. 3D), while the abundance of free fatty acids in serum was decreased in *Plin5*^*−/−*^ mice in the NAFLD model compared to WT littermates (cf. Suppl. Figs. 3E and 5A). Finally, only lysophosphatidylcholine (LPC) species 18:2 and 18:1 were different between *Plin5*^−/−^ and WT mice (Suppl. Fig. 5B).

### *Plin5* depletion alters the expression of the transcriptome in NAFLD by modulating the immune response and lipid metabolism

**Fig. 5:**
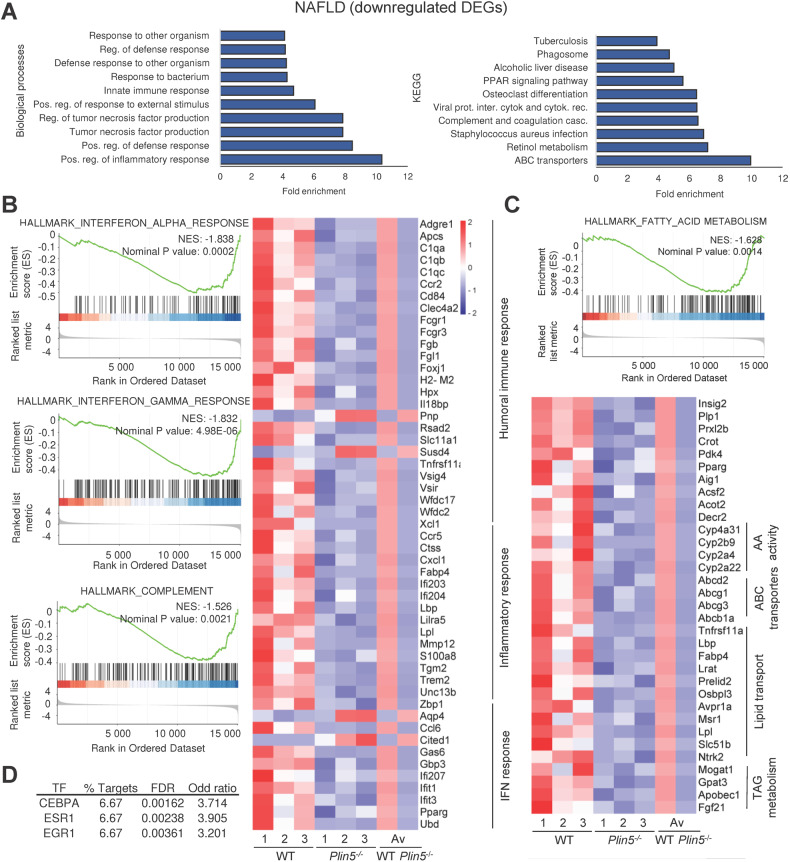
Deletion of *Plin5* induces hepatic reprogramming of the transcriptome during NAFLD. **A** Functional analyses of downregulated differentially expressed genes (DEGs) from WT-WD and *Plin5*^*−/−*^-WD of the NAFLD model. **B** Gene set enrichment analysis from WT-WD and *Plin5*^*−/−*^-WD of the NALFD model, indicating the reduced enrichment of key inflammatory pathways during NAFLD progression and its corresponding heatmap. **C** Enrichment in FA metabolism process from WT-WD and *Plin5*^*−/−*^-WD acetone-treated mice and its corresponding heatmap. **D** Analysis of transcription factors (TFs) targeting the set of downregulated DEGs from WT-WD and *Plin5*^*−/−*^-WD. (*n* = 3 mice per group). The average of the fold ratio for all WT and *Plin5*^*−/−*^ mice is represented in the heatmaps as average (Av).

Given that NAFLD and HCC are accompanied by transcriptional reprogramming, we next performed next-generation RNA sequencing (RNA-seq) to identify possible pathways in which *Plin5* plays a role during progression from NAFLD to HCC. Our analysis revealed 245 differentially expressed genes (DEGs) in *Plin5*^−/−^ mice compared to WT in the NAFLD model. Of these, 220 genes were downregulated and 25 upregulated. Functional analysis of the upregulated genes did not recover significant enrichment. However, the group of downregulated genes revealed that most genes participate in the immune response (Fig. 5A). Specifically, *Plin5*^−/−^ mice fed WD showed a significant down-regulation of genes implicated in the humoral immune response (*Adgre*, *C1q*, *Fcgr1*, *Fcgr3*, *Cd84*), acute inflammation (*Ccr2*, *Ccr5*, *Cxcl1*, *Mmp12*, *Trem2*), and interferon response (*Ccl6*, *Gas6*, *Gbp3*, *Ifi204*, *Ifi207*, *Slc11a1*, *Xcl1*). Furthermore, enrichment analysis revealed that the highest enriched pathways corresponded to interferon-α, interferon-γ and complement pathways (Fig. 5B). Fatty acid metabolism processes, arachidonic acid activity, ABC transporters, lipid transport, and TAG metabolic processes were found to be modulated in *Plin5*^*−/−*^ mice (Fig. 5C). A search of possible transcriptional factors that regulate DEGs yielded CCAAT/enhancer-binding protein-α (CEBPA) and estrogen receptor 1 (ESR1) as targets (Fig. 5D). These genes have functions during NAFLD, particularly regulating TAG synthesis in steatosis, gluconeogenesis, and lipid metabolism [24, 25]. In addition, the early growth response 1 (EGR1), which regulates insulin resistance and cholesterol biosynthesis, appeared to be significantly increased [26], while *Plin5*^−/−^ mice fed WD also had lower cholesterol levels in the NAFLD model.

Together, these results indicate that a lack of *Plin5* profoundly affects immunological processes and lipid homeostasis by targeting specific signaling pathways relevant in the pathogenesis of NAFLD.

In contrast, in the NAFLD-HCC model, a limited group of 32 DEGs were identified in the *Plin5*^*−/−*^ mice (Suppl. Fig. 6). Furthermore, comparison with the Human Protein Atlas (www.proteinatlas.org) [27] revealed that high expression of genes such as ribosomal protein S2 (*Rps2)*, Jupiter microtubule-associated homolog 2 (*Jpt2)*, purine nucleoside phosphorylase (*Pnp)*, and WD repeat domain 55 (*Wdr55)* was considered unfavorable for liver cancer prognosis, while high expression of glycine *N*-methyltransferase (*Gnmt)* was considered favorable. These data suggest that depletion of *Plin5* in the NAFLD-HCC model has only a small impact on the transcriptional signature.

### The loss of *Plin5* protects mice from activation of inflammatory signaling

**Fig. 6:**
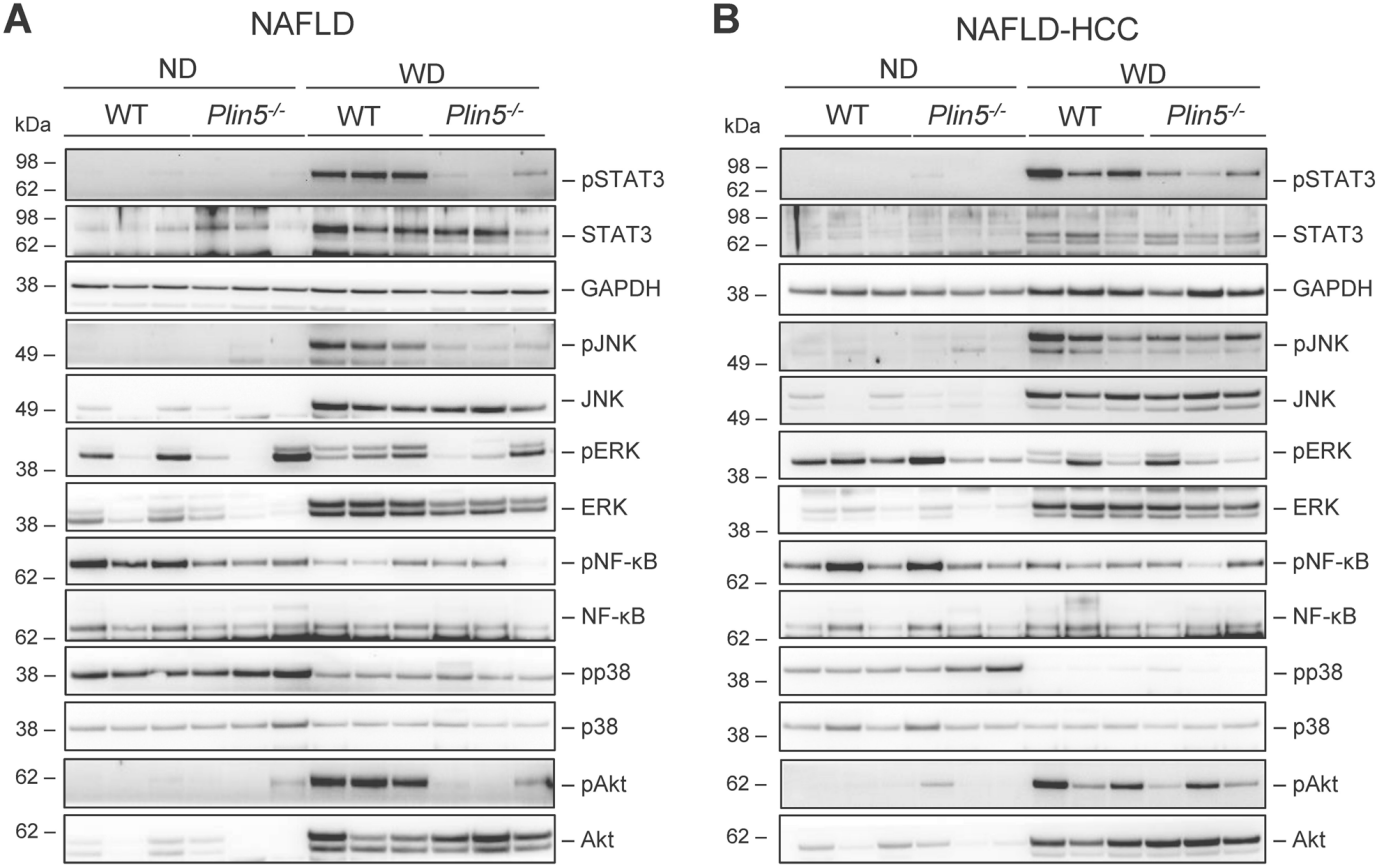
*Plin5* deficiency results in decreased STAT3 phosphorylation in livers of both NAFLD and NAFLD-HCC model. **A** Western blot analysis for detection of effector molecules of various inflammatory signaling pathways (pSTAT3, pJNK, pERK, pp38, pNF-κB, pAkt) in animals of the **A** NAFLD and **B** NAFLD-HCC model. Total proteins (STAT3, JNK, ERK, p38, NF-κB, Akt) and GAPDH expression served as controls to demonstrate equal protein loading.

We speculate that *Plin5* modulates relevant inflammatory pathways involved in carcinogenesis. Therefore, the activation of Janus kinase/signal transducer and activator of transcription 3 (JAK/STAT3) p38, mitogen-activated protein kinase kinase/extracellular signal-regulated kinase (MEK/ERK), nuclear factor-κB (NF-κB) and Jun N-terminal kinase (JNK) was analyzed by Western blot analysis. The levels of phosphorylated STAT3 (pSTAT3) were found to be significantly higher in controls fed WD than in controls fed ND. In contrast, WD-fed *Plin5*^*−/−*^ mice had lower pSTAT3 than WT mice in both models (Fig. 6 and Suppl. Fig. 7). Furthermore, phosphorylation of JNK (pJNK) was increased in animals that received WD. However, *Plin5*^*−/−*^ mice had lower levels of pJNK than WT littermates in the NAFLD model. Although the same tendency for reduced pJNK was observed in the NAFLD-HCC model, the difference between WT and *Plin5*^−/−^ mice was lower.

There was no significant difference in the level of ERK phosphorylation between any of the groups due to the high variation. In both models, the intensities of phosphorylated NF-κB (pNF-κB) dropped after WD feeding. However, there were no differences between WT and *Plin5*^−/−^ mice. Phosphorylation of p38 (pp38) was reduced after 30 weeks of WD in both models compared to ND-fed mice independent of genotype. Lastly, it was found that activation of Akt (pAkt) increased in WD-fed WT animals in both models. Interestingly, while WT mice in the NAFLD model had higher levels of pAkt than *Plin5*^*−/−*^ mice, pAkt levels were comparable in the NAFLD-HCC model.

Collectively, the data indicate that loss of *Plin5* is associated with reduced levels of important inflammatory markers (pSTAT3, pJNK), suggesting that deletion of *Plin5* protects against inflammation in the pathology of NAFLD and NAFLD-HCC.

## Discussion

Consumption of high-caloric and high-fat foods is an important factor in the development of steatohepatitis and NAFLD. Moreover, NAFLD is associated with an increased risk of HCC development [28]. During the development of NAFLD and HCC, perilipins have been recognized as key proteins involved in lipid accumulation. In particular, the role of *Plin5* in the liver has been intensively studied [10–13]. Although different models have been recapitulated to establish NAFLD in *Plin5*^*−/−*^ mice, the results seem to vary depending on the diet used [10–13]. Furthermore, no NAFLD-HCC models have previously been reported to discover the roles of *Plin5*.

Here, we demonstrate for the first time that the loss of *Plin5* prevents liver tumorigenesis by modulating inflammatory signaling. Our results show that in WD feeding, *Plin5*^*−/−*^ mice exhibited reduced hepatosteatosis, confirming previous reports showing less hepatic fat deposition in *Plin5*^*−/−*^ mice fed a HFD [10, 11]. Interestingly, the loss of *Plin5* is associated with less aggravated liver injury only in the NAFLD model but not in the NAFLD-HCC model (Fig. 2). This finding is similar to the results obtained in a previous model in our laboratory [11], but different from the results reported by others [10, 12]. We speculate that differences in diet composition could be the cause of this contradiction [29–31].

Furthermore, we show that *Plin5* depletion did not improve glucose tolerance, which is also contrary to a previous report that found *that Plin5*^*−/−*^ mice fed with a HFD had a better glucose response than WT mice [23]. The addition of glucose/fructose to drinking water has been established to worsen the effects on glucose tolerance in WT mice [32]. Thus, we speculate that the addition of glucose/fructose to drinking water blunted the effects caused by the genetic deletion of *Plin5*.

We demonstrated that *Plin5*^*−/−*^ mice present differential hepatic lipid accumulation mostly by regulating LD formation and size, since their distribution was different in our NAFLD and NAFLD-HCC models (Fig. 3). At the molecular level, we found changes in the expression of genes involved in FAO under ND. Interestingly, the absence of *Plin5* is accompanied by a reduction of PGC-1α in WD-fed mice. In line, PLIN5 has been recognized as a transcriptional co-regulator of PGC-1α and a strong regulator of mitochondrial function [9]. Therefore, we hypothesize that in the absence of PLIN5, a decrease in PGC-1α and possibly its downstream targets leads to reduced levels of CPT1 in *Plin5*^*−/−*^ mice in NAFLD. We further speculate that the combination of diet and the addition of fructose to drinking water could lead to modified FAO. Consistent with this assumption, the addition of fructose to a HFD has been shown to increase liver malonyl-CoA, therefore inhibiting CPT1, resulting in decreased FAO and the development of metabolic dysregulation [33]. In contrast, WT and *Plin5*^*−/−*^ mice exposed to the carcinogen (i.e., DMBA) showed similar levels of CPT1. The difference observed in *Plin5*^*−/−*^ mice between the NAFLD and NAFLD-HCC models can be explained by the requirements of the cells to satisfy their energy supply. Fatty acids can provide energy for cancer cells, and mitochondrial FAO yields more ATP per mole than the oxidation of biomolecules such as glucose or amino acids [34]. Therefore, an increase in FAO driven by higher levels of CPT1 is a logical mechanism to compensate for energy requirements.

When analyzing serum lipid composition, we found a differential ratio of SM constituted of very long chain fatty acids in *Plin5*^*−/−*^ mice (Fig. 4D, Suppl. Table 1). In line, previous studies indicate that SM 18:1/18:0, 18:1/20:0, 18:1/22:0 and 18:1/24:0 correlated with obesity, insulin resistance, liver function, and lipid metabolism [35].

By analyzing the transcriptional signature of *Plin5*^*−/−*^ mice subjected to a WD, we identified a set of downregulated genes related to inflammation and lipid metabolism (Fig. 5). These pathways coordinate the progression of NAFLD by regulating key inflammatory-acting genes such as *Ccl6*, *Gas6*, *Gbp3*, *Slc11a1*, and others that are involved in the IFN response and are known to drive NAFLD [36]. When analyzing genes related to lipid metabolism, we identified four clusters of genes that regulate fatty acid homeostasis. Interestingly, targets that regulate the activity of arachidonic acid are predicted to have monooxygenase activity. The incorporation of oxygen by cytochrome P450 members into arachidonic acid leads to the formation of epoxy- and hydroxyl-fatty acids (i.e., hydroxyeicosatetraenoic acids) [37]. The latter group has been studied for its inflammatory properties during NAFLD [38]. Within this signature, we also found reduced expression of genes that regulate lipid transport, such as fatty acid binding protein 4 (*Fabp4*), previously associated with increased lipid uptake and liver fatty infiltration in patients with NAFLD [39]. These data pinpoint how loss of *Plin5* can partially protect against NAFLD by regulating the immune response, the inflammatory response, and lipid metabolism.

According to the reduction in fibrosis in *Plin5*^*−/−*^ mice (Fig. 1C), we found that liver expression of lipoprotein lipase (*Lpl*) and lecithin retinol acyltransferase (*Lrat*) decreased in *Plin5*^−/−^ mice fed WD. Repression of *Lpl* in hepatic stellate cells significantly reduces fibrosis in a NASH model [40]. On the other hand, activated hepatic stellate cells have upregulated levels of *Lrat* and loss of *Lrat* expression is associated with reduced fibrosis [41]. Another study demonstrated that *Lrat* depletion reduces susceptibility to hepatocarcinogenesis [42]. These findings confirm a study showing reduced liver fibrosis, intrahepatic TAG content, and fatty acid synthesis in *Plin5*^*−/−*^ mice fed HFD [13]. Similarly, a previous study demonstrated that inhibition of the LPL/FABP4/CPT1 axis can effectively prevent the progression of NASH to liver cancer [43]. Although future in vitro experiments would help to elucidate the molecular mechanism involved in this process, it is possible to argue that *Plin5*^*−/−*^ partially regulates their antifibrotic role by down-regulating *Lpl* and *Lrat*.

Analysis of the transcriptome signature of the *Plin5*^*−/−*^ mice in the NAFLD-HCC model resulted in only 32 DEGs (Suppl. Fig. 6). Thus, we speculate that carcinogenesis hinders a more extensive modulation of the transcriptional signature observed in the NAFLD model in the absence of *Plin5*. Finally, when comparing different pathways altered in NAFLD and NAFLD-related carcinogenesis, we identified several important effector molecules that have been previously reported in various models of NAFLD and HCC, including STAT3, p38, JNK, and Akt [44]. In particular, *Plin5* deficiency was shown to reduce STAT3 phosphorylation in *Plin5*^*−/−*^ WD mice compared to WT mice in both models. Interestingly, while STAT3 phosphorylation is completely absent in *Plin5*^*−/−*^ mice in the NAFLD model, it is only slightly reduced in the NAFLD-HCC model, possibly due to additional influence of DMBA on inflammatory signaling pathways. The importance of this finding lies in the fact that STAT3 has been reported by many others as one of the main drivers of fibrosis [45–47]. This finding is complementary to a previous study by us showing that the IL-6/STAT3 axis regulates PLIN5 expression and indicates that there is a feedback loop between STAT3 and PLIN5 [19]. Another interesting finding is the activation of JNK after WD in WT mice in both models, while JNK phosphorylation was significantly reduced in the NAFLD model after loss of *Plin5*. Taking into account that JNK mediates steatosis and glucose intolerance, this finding makes PLIN5 a clinically relevant target [48]. In this context, it should be noted that the JNK inhibitor JM-2 significantly protected mouse livers from HFD-induced inflammation, lipid accumulation, fibrosis, and apoptosis [49], which is consistent with our findings.

**Fig. 7:**
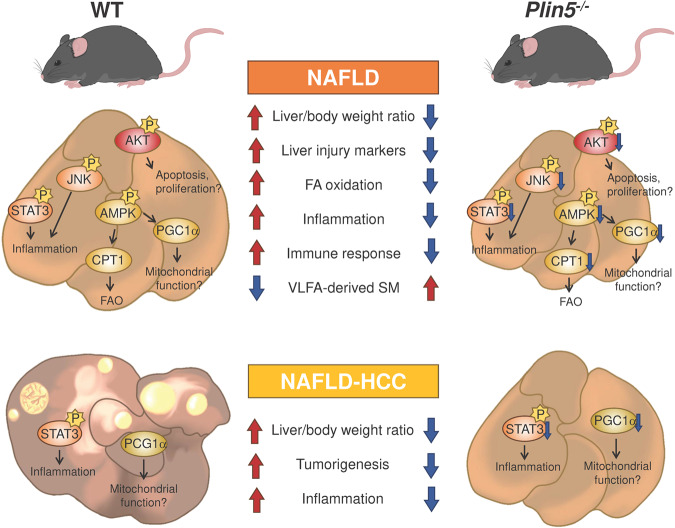
Schematic representation of the effects of *Plin5* deficiency in NAFLD and NAFLD-HCC models. Abbreviations used are. AKT protein kinase B, CPT1 carnitine palmitoyltransferase 1, JNK Jun N-terminal kinase, PGC-1α peroxisome proliferator‐activated receptor γ coactivator 1α, SM sphingomyelins, STAT3 signal transducer and activator of transcription 3; VLFA very long chain fatty acids. This illustration was created using Procreate, Photoshop CS and Microsoft Power Point (version 365).

We recapitulated the most significant findings of our NAFLD and NAFLD-HCC and the roles of *Plin5* in Fig. 7. Collectively, we demonstrated that loss of *Plin5* protects against worsening of NAFLD by regulating inflammatory signaling, mitochondrial function, and lipid metabolism, while in the NAFLD-HCC model, suppression of pSTAT3 favors a milder inflammatory response thus preventing severe liver injury.

More studies will be necessary to elucidate the molecular mechanisms by which *Plin5* promotes the switch from NAFLD to HCC and therapeutic options to target HCC development. In particular, more studies are needed to explore the mechanistic insights of PLIN5 activity and identify the cell types responsible for mediating these effects.

## Materials and methods

### Animals and induction of liver tumors

Male C57BL/6J mice WT and *Plin5* knockout (*Plin5*^*−/−*^) were obtained from the Institute of Laboratory Animal Science of the Medicine Faculty of RWTH Aachen University as previously described [11] and kept under controlled conditions of temperature (20 °C) and humidity (50%) in a 12:12 h light-dark cycle.

#### Model

WT and *Plin5*^*−/−*^ mice were grouped randomly into different groups, and treated as previously described [20] with slight modifications. Briefly, 4–5 days old male mice were treated with 50 µL of 0.5% 7,12-dimethylbenz[a]anthracene (DMBA) or acetone as control, applied to the dorsal surface of the neck area. Once absorbed, the pups were returned to their mothers for three weeks until weaning. After the weaning period, mice were randomly assigned to a specific diet (6–9 mice per group, calculated with GPower3.1). Mice treated with acetone represented the NAFLD model, while mice treated with DMBA served as the model for NAFLD-induced HCC [20].

#### Diets

All animals were fed ad libitum for 30 weeks. The control groups were fed with a normal diet (ND) (V1534, ssnif Spezialdiäten GmbH, Soest, Germany) composed of 58% carbohydrates, 33% protein, and 9% fat. The other four experimental groups were fed with a WD (D17010102, Research Diets, Inc., New Brunswick, NJ, USA), composed of 40% fat, 20% fructose, and 2% cholesterol. Mice on the WD also had ad libitum access to water supplemented with fructose 55% (w/v) and glucose 45% (w/v) changed every second week. Body weight was measured once a week. After 30 weeks the mice were sacrificed during the light period, under inhalation anesthesia using isoflurane (Forene, Abbott, Wiesbaden, Germany).

All animals used in this study received humane care. All protocols were in full compliance with the guidelines for animal experimentation and were approved by the institutional German Animal Care Committee (LANUV, Recklinghausen, Germany, permit Az.:81-02.04.2019.A366).

### Liver histology

After dissection, small sections of the liver were fixed in 4% paraformaldehyde (Otto Fischar GmbH, Saarbrücken, Germany) for 24 h. Dehydration was carried out using a fully automated tissue processor at the Interdisciplinary Center for Clinical Research (IZKF) located at the RWTH University Hospital Aachen, after which the tissue was embedded in paraffin and sections were cut to a thickness of 5 µm. The slides were dried at 37 °C before staining. Hematoxylin and eosin (HE) and Sirius red staining were performed using standard procedures and slides were scanned with the NanoZoomer SQ digital slide scanner C13140-21 (Hamamatsu Photonics, Hamamatsu, Japan). NDP.view2 viewer software (Ver. 2.9.25) was used for visualization (U12388-01, Hamamatsu Photonics). A blinded pathologist performed the histological scoring of murine steatosis. The guidelines used for the scoring were adopted from NAFLD/NASH in humans [50].

### Computed tomography (CT)

CT scans were performed as previously described [20].

### Oil Red O staining

The embedded frozen liver tissues were cut into 10-μm sections using a Leica cryostat. The slides were washed with phosphate buffered saline and fixed in 4% paraformaldehyde for 2 min. The 0.3% Oil-red O solution was filtered before use and applied on each slide for 5 min at room temperature. The slides were washed twice with distilled water for 10 min. Hematoxylin was used for counterstaining, and the slides were washed five times with distilled water, and finally mounted. The images were acquired with a NIKON Eclipse 80i inverted microscope (Nikon, Tokyo, Japan).

### Lipid droplet size measurements

The size of the LD was calculated as described in [51]. For each analysis, three mice from each group were used and five fields of each image were analyzed.

### Glucose tolerance test

At 10 and 20 weeks after the start of the diet, the mice were subjected to a glucose tolerance test. Before the test, the mice were fasted for 5 h and baseline glucose levels were measured in tail-tip blood drops with a blood glucose glucometer (Accu-Chek Instant, Roche, Basel, Switzerland). Mice were injected intraperitoneally with D-glucose (G8769, Sigma Aldrich, Taufkirchen, Germany) at a dose of 2 mg/kg. Glucose levels were assessed at 15, 30, 60 and 120 min after injection.

### Serum analysis

Whole blood was collected from the heart and serum was separated by centrifugation in a Microvette® 500 serum gel extraction tube (Sarstedt, Nümbrecht, Germany) at 7000 × *g* for 5 min. Liver enzymes, including alkaline phosphatase, alanine aminotransferase, aspartate aminotransferase, lactate dehydrogenase, and biochemical parameters such as TAGs and cholesterol, were measured in serum using standard laboratory techniques. Blinded analysis by a technician was performed on the samples. For lipidomic analysis, 50 µL of serum was used.

### Transmission electron microscopy

Liver tissue pieces were treated as previously described [11]. The images were acquired at magnifications of 6000× and 10,000×.

### RNA isolation, cDNA synthesis, and RT-qPCR

All methods were performed as described previously [52]. Primer sequences used for RT-qPCR are given in Suppl. Table 2.

### Primary analysis of RNA sequencing

Triplicate biological replicates were performed for each group. cDNA for libraries was produced using the 36x Colibri 3’ mRNA library preparation kit (Invitrogen, Thermo Fisher Scientific, Meerbuch, Germany) with an input of 500 ng of total RNA and 1% of denatured and diluted PhiX. The library was essentially prepared as described [53]. Quality control was carried out with FastQC v0.11.9. The reads were assigned to the mouse reference genome (mm10.GRCm38.GCA_000001635.2) using STAR [54] (version 2.7.3a) with the following settings: --outFilterScoreMinOverLread 0.25 --outFilterMatchNminOverLread 0.25 --alignSoftClipAtReferenceEnds “No” to optimize short read mapping. Only uniquely mapped reads were counted over gene features annotated from Ensembl (Mus_musculus.GRCm38.100.) using featureCounts [55] (version 2.0.0) with parameter --primary.

### Differential gene expression analysis

Differential expression analysis was performed on each pairwise comparison of genotype, treatment, and diet. The transformed rlog counts (rlog) were used for the analysis of the principal components and the average grouping of samples according to the Euclidean distance using the pheatmap function of the R pheatmap package [56]. The DESeq2 package [57] in R was used to calculate statistical fold changes using the LFC shrinkage apeglm package [58]. Differentially expressed genes were considered statistically differentially expressed with an adjusted *p*-value < 0.05 and (log2-fold change) > 1.

### Functional enrichment analysis

Functional enrichment analyzes were performed with the clusterProfiler package v3.16.1 [59]. The annotation of gene ontology consists of three parts: biological process (BP), cellular component (CC), and molecular function (MF). Gene set enrichment analysis is a method used to determine if a pre-defined set of genes is enriched in a ranked list of genes. All genes were classified by decreasing log2-fold change and the gene sets used were hallmarks relevant to *Mus musculus* obtained from the MSigDBr package [60] in R.

Overrepresentation analysis was performed separately for significantly upregulated (log2FC > 0) and downregulated (log2FC < 0) genes using function enrichGO. Comparison of gene clusters (up- and downregulated genes) and functional profiles was done using the compareCluster function. Statistically significant pathways were defined with a Benjamini-Hochberg adjusted *p*-value < 0.05. Heat maps were plotted using http://www.bioinformatics.com.cn/srplot, an online platform for data analysis and visualization.

### Liver protein extraction, SDS-PAGE, and Western blot analysis

Protein extracts were prepared with slight modifications as described previously [61]. In summary, liver tissues were homogenized in a Mixer Mill MM400 homogenizer in RIPA buffer (50 mmol/l Tris-HCl, pH 8.0; 0.25 mol/l NaCl; 5 mmol/l EDTA) without Triton X-100 supplemented with the Complete™-mixture of proteinase inhibitors (Roche) and the Phosphatase Inhibitor Cocktail 2 (1:100, Sigma-Aldrich). Homogenates were centrifuged at 6000 × *g* for 15 min at 4 °C twice to remove the lipid layer. Triton X-100 was added to protein extracts at a final concentration of 1% (v/v) and centrifuged three times at 12,000 × *g* for 15 min at 4 °C. Equal amounts of proteins (75 µg) were diluted in Nu-PAGE™ LDS electrophoresis sample buffer (Thermo Fisher Scientific) with 50 mM dithiothreitol as reducing agent. The samples were incubated at 85 °C for 10 min and the proteins were separated in 4–12% Bis-Tris gels (Invitrogen) in MES running buffer. Proteins were blotted onto Amersham™ Protran® nitrocellulose membranes with a 0.45 µm pore size (Merck, Darmstadt, Germany). To avoid unspecific binding, membranes were blocked in TBS-T (10 mM Tris/HCl, 150 mM NaCl, 0.1% (v/v) Tween 20, pH 7.6) containing 5% (w/v) non-fat milk powder. The membranes were subsequently incubated overnight at 4 °C with the antibodies listed in the Suppl. Table 3. For visualization, membranes were incubated with the listed secondary antibodies coupled to horseradish peroxidase antimouse, antirabbit, or antigoat IgG (Invitrogen) and the SuperSignal chemiluminescent substrate (Pierce, Bonn, Germany) using the iBright imaging system (Thermo Fisher Scientific). To quantify Western blot signals, we first converted the original digitalized images of the Western blots into 8-bit images using the image processing and analysis software ImageJ. Next, the images were inverted to black and white and adjusted the gray-scale threshold for the black and white image. We then framed the signals within the region of interest and exported the measured signal intensity to a Microsoft Excel spreadsheet. Finally, we determined the relative quantification of values by calculating the ratio of the net band to the net loading control, which could be a house-keeping gene or an unphosphorylated protein.

### Lipid extraction and analysis of lipidomics

Liver samples were homogenized in 500 µl methanol using a Precellys tissue homogenizer (Berlin Technologies, Montigny-le Bretonneux, France). Homogenized samples were transferred to a glass vial. Lipids were extracted according to the method of Bligh and Dyer using 1:1 chloroform and methanol (1.5 ml each). The samples were mixed for 30 min at 700 rpm and room temperature using a horizontal shaker. Water was added, samples were shaken for 10 min at 700 rpm and room temperature and subsequently centrifuged for 7 min at 3500 rpm and room temperature to separate the lipid-containing organic from the aqueous phase. One ml of the lower (organic) phase was withdrawn using a glass syringe (Hamilton) and extraction was repeated by adding 1 ml of chloroform, vigorous shaking and centrifugation. The organic phase was again withdrawn and the organic phases of one sample were combined, evaporated to dryness, and dissolved in 100 µl chloroform.

Ten µl of each sample were automatically sprayed onto a normal phase high performance thin layer chromatography plate. Phospholipid classes were separated by chloroform/ethanol/water/triethylamine (30:35:7:35, by vol.) or n-hexane/diethyl ether/glacial acetic acid (80:15:1, by vol.).

### Statistics

Statistical analysis and graphics were performed using GraphPad Prism 8 software (version 8.4.2, San Diego, CA, USA). The significance of the differences is presented as means ± SD, except for the area under the curve (AUC) for the GTT where the results are presented as means ± SE. The *n*, significance values and test performed are indicated in the legends of each figure. The probability values given are *p < 0.05, **p < 0.01, ***p < 0.001 and ****p < 0.0001 respectively. For lipid analysis, we preformed multiple t-tests and corrected for multiple comparisons using the Holm-Sidak methods. The figures were assembled using Microsoft Excel (version 2307) and Power Point (version 2307).

### Supplementary information

Supplements

Supplementary Figure 1

Supplementary Figure 2

Supplementary Figure 3

Supplementary Figure 4

Supplementary Figure 5

Supplementary Figure 6

Supplementary Figure 7

Original Data File

## Data Availability

The data supporting the findings of this study are available from the corresponding authors, AA and RW, upon reasonable request. The RNA-seq data supporting the findings of this study were submitted to Gene Expression Omnibus (GEO, https://www.ncbi.nlm.nih.gov/geo/) under access. no. GSE242852.
