# Supplementary material for: Perilipin 5 deletion protects against nonalcoholic fatty liver disease and hepatocellular carcinoma by modulating lipid metabolism and inflammatory responses
Source: Cell Death Discov. 2024 Feb 22;10:94. doi: 10.1038/s41420-024-01860-4 (PMC10884415; doi:10.1038/s41420-024-01860-4)
Supplement: Supplementary file 1 — Supplements [file 41420_2024_1860_MOESM1_ESM.docx]

**Supplementary Materials**

**Perilipin 5 deletion protects against nonalcoholic fatty liver disease and hepatocellular carcinoma by modulating lipid metabolism and inflammatory responses**

Paola Berenice Mass-Sanchez^1^, Marinela Krizanac^1^, Paula Štancl^2^, Marvin Leopold^3^, Kathrin Engel^3^, Eva Miriam Buhl^4^, Joseph van Helden^5^, Nikolaus Gassler^6^, Jürgen Schiller^3^, Rosa Karlić^2^, Diana Möckel^7^, Twan Lammers^7^, Steffen K. Meurer^1^, Ralf Weiskirchen^1^* and Anastasia Asimakopoulos^1^*

**Supplementary Tables**

Suppl. Table 1: Significances of sphingomyelins in the liver and serum.

Suppl. Table 2: List of primers used in this study.

Suppl. Table 3: List of antibodies used in this study.

**Supplementary Figures**

Suppl. Figure 1: PLIN5 increases during NAFLD and NAFLD-HCC.

Suppl. Figure 2: Impairment in glucose tolerance is observed after 10 weeks of diet in WT and *Plin5*^-/-^ mice.

Suppl. Figure 3: Hepatic lipidomic composition is altered during NAFLD and NAFLD-HCC.

Suppl. Figure 4: The phosphatidylinositol profiles are not different in WT and *Plin5^-/-^* mice.

Suppl. Figure 5: Free fatty acids in serum and lysophosphatidylcholines ratios are not altered after the loss of *Plin5*.

Suppl. Figure 6: The loss of *Plin5* does not drive transcriptional alterations in the NAFLD-HCC model.

Suppl. Figure 7: Statistics to Western blot analysis depicted in Figure 6.

**Suppl. Table 1: Significances of changes of selected sphingomyelin species in the liver and serum.**

| **Comparison** | **Species** | ***p-*value** |
| --- | --- | --- |
| **Liver** | | |
| WT ND Acetone *vs*. WT WD Acetone | SM d18:1/22:0 | 0.0005 |
|  | SM d18:1/18:0 | 0.0012 |
| *Plin5*^-/-^ ND Acetone *vs.* *Plin5*^-/-^ WD Acetone | SM d18:1/24:0 | 0.0003 |
|  | SM d18:1/22:1 | 0.0008 |
| *Plin5^-/-^* ND DMBA *vs*. *Plin5^-/-^* WD DMBA | SM d18:1/22:1 | <0.0001 |
| **Serum** | | |
| WT ND Acetone *vs*. WT WD Acetone | SM d18:1/18:0 | <0.0001 |
|  | SM d18:1/18:1 | 0.0001 |
| **WT WD Acetone *vs. Plin5^-/-^* WD Acetone** | **SM d18:1/22:1** | **0.0004** |
|  | **SM d18:1/22:0** | **0.001** |
|  | **SM d17:1/24:1** | **0.0029** |
|  | **SM d18:1/16:0** | **0.0045** |
| *Plin5^-/-^* ND Acetone *vs*. *Plin5^-/-^* WD Acetone | SM d18:1/18:1 | <0.0001 |
|  | SM d18:1/22:1 | <0.0001 |
| WT ND DMBA *vs.* WT WD DMBA | SM d18:1/18:1 | 0.0001 |
|  | SM d17:1/24:1 | 0.0007 |
|  | SM d18:1/24:2 | 0.0014 |
|  | SM d18:1/20:0 | 0.0031 |
| **WT ND DMBA *vs.* *Plin5^-/-^* ND DMBA** | **SM d18:1/20:0** | **<0.0001** |
|  | **SM d18:1/24:2** | **0.00086** |
|  | **SM d18:1/24:0** | **0.002954** |
|  | **SM d18:1/16:1** | **0.004071** |
| *Plin5^-/-^* ND DMBA *vs.* *Plin5^-/-^* WD DMBA | SM d18:1/20:0 | <0.0001 |
|  | SM d18:1/18:1 | <0.0001 |
|  | SM d18:1/24:2 | 0.0012 |
|  | SM d17:1/24:1 | 0.0027 |
| WT ND Acetone *vs.* WT ND DMBA | SM d18:1/18:0 | 0.000115 |
| WT WD Acetone *vs.* WT WD DMBA | SM d18:1/24:0 | 0.0025 |
|  | SM d18:1/16:0 | 0.0037 |
|  | SM d18:1/24:1 | 0.0053 |
| *Plin5^-/-^* ND Acetone *vs.* *Plin5^-/-^* ND DMBA | SM d18:1/20:0 | <0.0001 |
|  | SM d18:1/24:2 | 0.0008 |
|  | SM d18:1/24:0 | 0.0016 |

Note: Significant differences between WT and *Plin5*^-/-^ mice in the different models are marked in bold. Up-regulated SM species in *Plin5*^-/-^ mice *vs.* WT mice are indicated in red letters, and blue letters indicate down-regulated levels of SM species in *Plin5*^-/-^ mice *vs*. WT mice.

**Suppl. Table 2: List of primers used in this study.**

| **Gene** | **Accession Nr** | **Primers** |
| --- | --- | --- |
| *Gapdh* | XM_001473623 | For: 5’-ACT GCC ACC CAG AAG ACTG-3’  Rev: 5’-CAC CAC CCT GTT GCT GTA G-3’ |
| *Cpt1* | NM_153679 | For: 5’-AGAGAAGCCTGCCAGTTTGTGAGA-3’  Rev: 5’-TGTACAGTGCAAAGAGGTGACGGT-3’ |
| *Scad* | NM_007383.3 | For: 5’-AGGTCCTGGAGGTCTGTGC-3’  Rev: 5’-CAGTCCCGAACACCGAGA-3’ |
| *Mcad* | NM_007382.5 | For: 5’-AGTACCCTGTGGAGAAGCTGAT-3’  Rev: 5’-TCAATGTGCTCACGAGCTATG-3’ |
| *Lcad* | NM_007381.4 | For: 5’-GCTTATGAATGTGTGCAACTCC-3’  Rev: 5’-CCGAGCATCCACGTAAGC-3’ |
| *Acox2* | [NM_053115.2](https://www.ncbi.nlm.nih.gov/nucleotide/NM_053115.2?report=genbank&log$=nucltop&blast_rank=6&RID=74VYV0HC013) | For: 5’-GACGGTCCTGAACGCATTT-3’  Rev: 5’-CATTCATGGCAATACCATGTAAGTT-3’ |

**Suppl. Table 3: List of antibodies used in this study.**

| **Antibody** | **Cat. No** | **Company** | **Size (kDa)** | **Dilution** |
| --- | --- | --- | --- | --- |
| PLIN5 | 26951-1-AP | Proteintech, Manchester, UK | 51 | 1:1,000 |
| GAPDH | sc-32233 | Santa Cruz Biotech., Santa Cruz, CA, USA. | 38 | 1:1,000 |
| CPT1 | sc-393070 | Santa Cruz Biotech. | 86 k | 1:1,000 |
| PGC1A | ST1202 | Sigma-Aldrich, Taufkirchen, Germany | 113 | 1:1,000 |
| pAMPK | 2531S | Cell Signaling Technology, Leiden, The Netherlands | 62 | 1:1,000 |
| AMPK | 2532S | Cell Signaling | 62 | 1:1,000 |
| pSTAT3 | #9134 | Cell Signaling | 84-91 | 1:1,000 |
| STAT3 | sc-8019 | Santa Cruz Biotech. | 84-91 | 1:1,000 |
| pSAPK/JNK | #4668 | Cell Signaling | 46/54 | 1:1,000 |
| SAPK/JNK | #9252 | Cell Signaling | 46/54 | 1:1,000 |
| pERK1/2 | #9102 | Cell Signaling | 42/44 | 1:1,000 |
| ERK1/2 | #9101 | Cell Signaling | 42/44 | 1:1,000 |
| pNF-κB | #3031 | Cell Signaling | 65 | 1:1,000 |
| NF-κB | sc-8008 | Santa Cruz Biotech. | 65 | 1:1,000 |
| pp38 | 612289 | BD Biosciences, Heidelberg, Germany | 38 | 1:1,000 |
| p38 | 612281 | BD Biosciences | 38 | 1:1,000 |
| pAkt | #4060 | Cell Signaling | 62 | 1:1,000 |
| Akt | #4691 | Cell Signaling | 62 | 1:1,000 |
| goat anti-mouse IgG (H+L), HRP | #31430 | Invitrogen, Thermo Fisher Scientific, Meerbusch, Germany | - | 1:5,000 |
| goat anti-rabbit IgG (H+L), HRP | #31460 | Invitrogen | - | 1:5,000 |
| mouse anti-goat IgG (H+L), HRP | #31400 | Invitrogen | - | 1:5,000 |


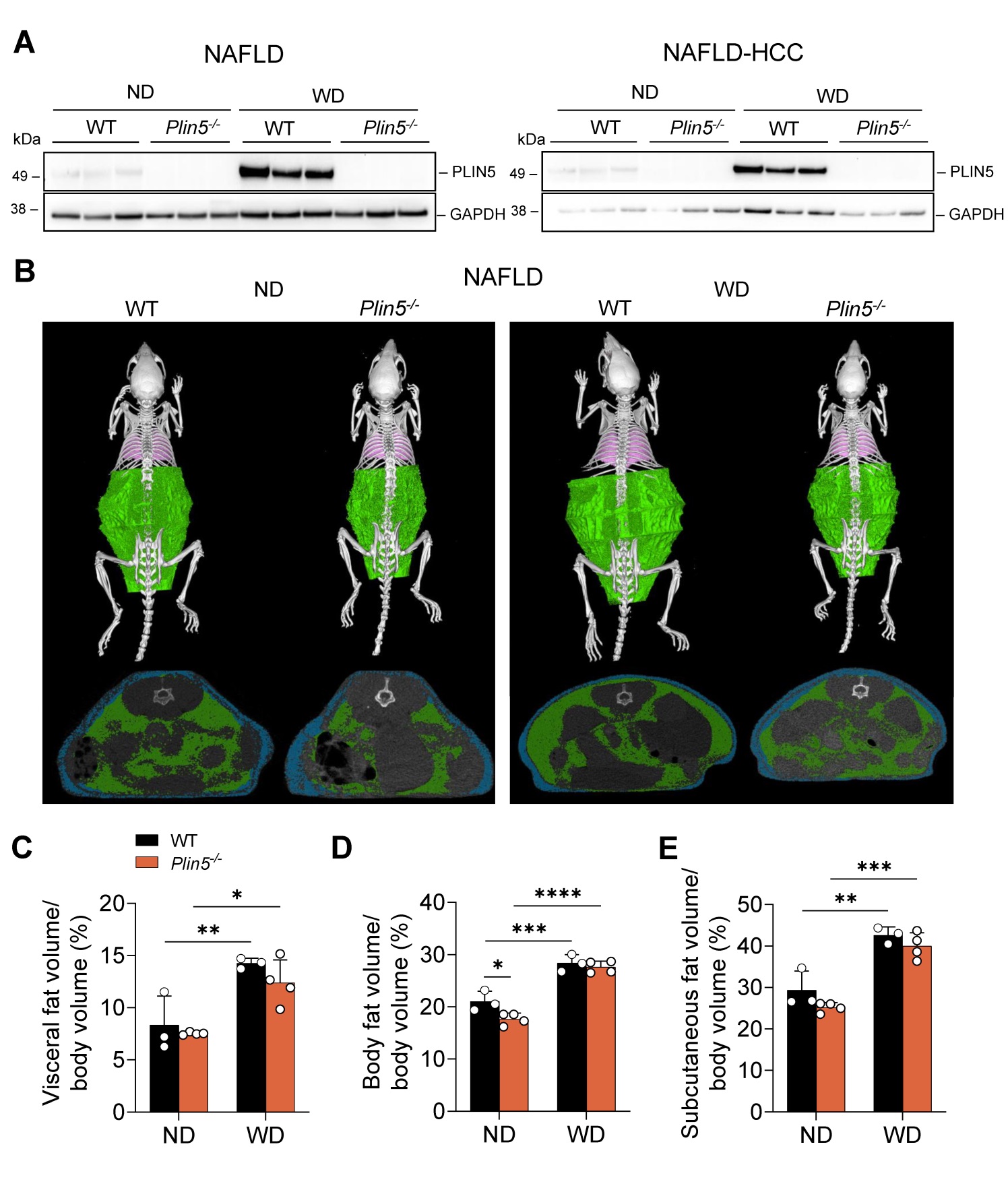


**Suppl. Figure 1: PLIN5 increases during NAFLD and NAFLD-HCC.** **(A)** Expression of PLIN5 levels in mice (n=3 in each group) fed with normal (ND) or Western diet (WD) in the NAFLD and NAFLD-HCC models evaluated by Western blot analysis. GAPDH expression served as a control to demonstrate equal protein loading. **(B)** Computerized tomography scans of representative WT and *Plin5^-/-^* mice fed ND or WD in the NAFLD model. **(C-E)** Measurement of fat mass calculated from CT scans of **(C)** visceral fat volume to body volume, **(D)** body fat volume to body volume, and **(E)** percentage of subcutaneous fat. Data represent mean ± SD. All data were analyzed by two-way ANOVA using the Tukey post-test. * *p* <0.05, ** *p* <0.01, *** *p* <0.001, **** *p* <0.0001.


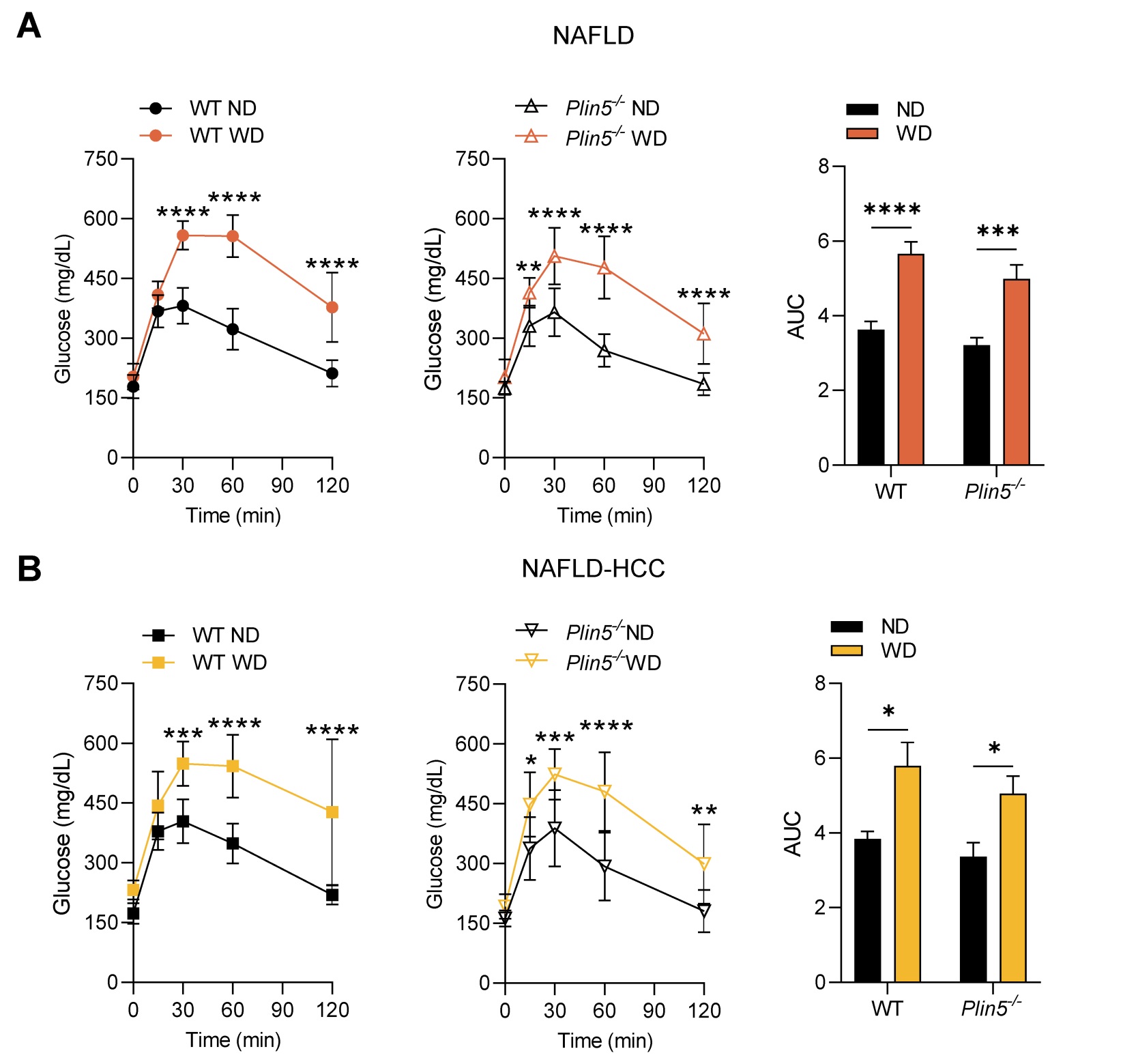


**Suppl. Figure 2: Impairment in glucose tolerance is observed after 10 weeks of diet in wild-type and *Plin5*^-/-^ mice.** Glucose tolerance tests were performed 10 weeks after subjecting the animals to the normal (ND) or Western diet (WD) after treatment with **(A)** acetone (n=8) or **(B)** DMBA (n=6-8). AUC: area under the curve. Data represent mean ± SD except in the calculation of AUC where data were represented as mean ± SE. All data was analyzed by two-way ANOVA using Tukey post-test. * *p* <0.05, *** *p* <0.001, **** *p* <0.0001.


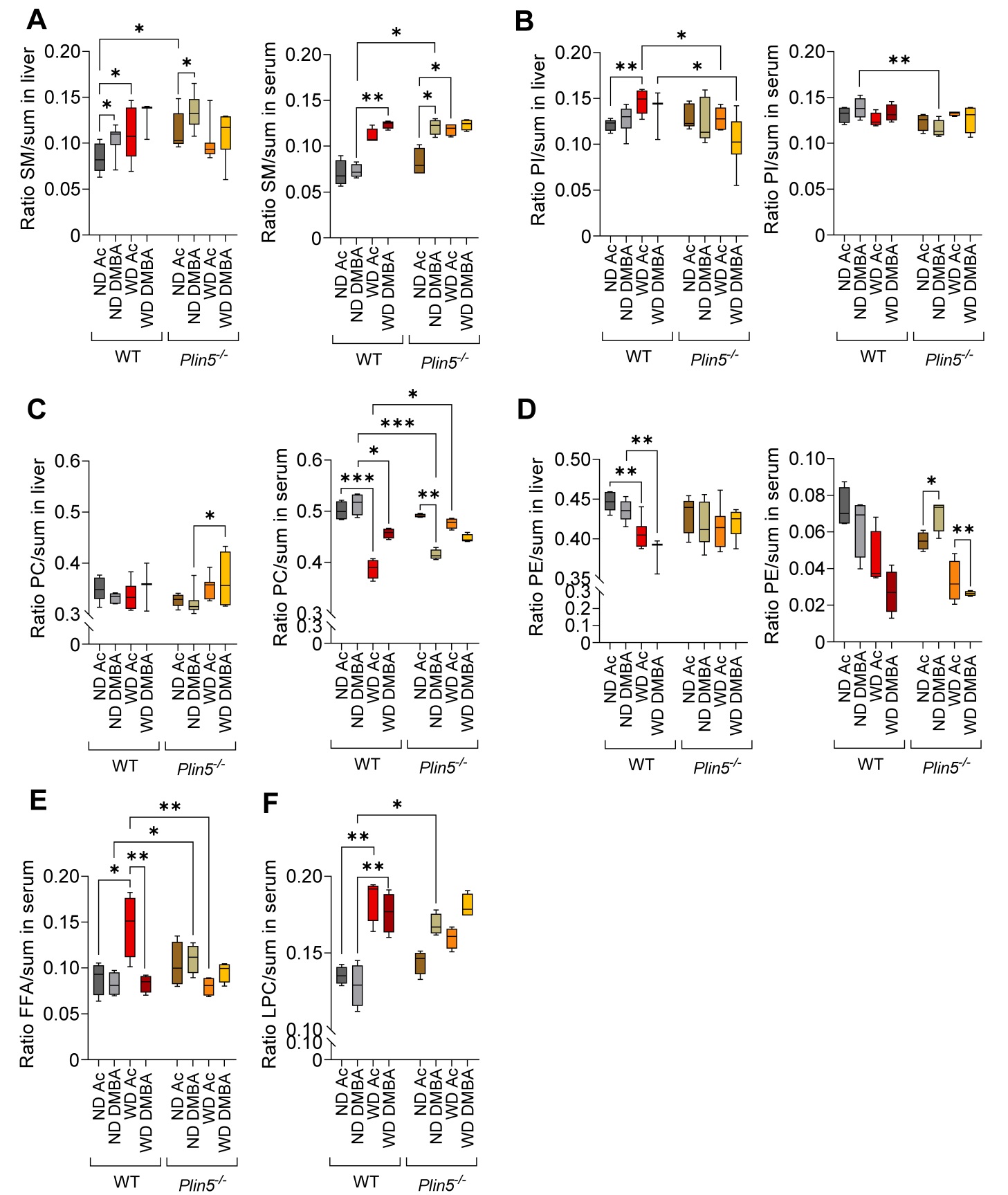


**Suppl. Figure 3: Hepatic lipidomic composition is altered during NAFLD and NAFLD-HCC.** **(A-D)** Sum of the relative ratio in the liver and serum of **(A)** sphingomyelins (SM), **(B)** phosphatidylinositol (PI), **(C)** phosphatidylcholine (PC) and **(D)** phosphatidylethanolamine (PE) of WT and *Plin5^-/-^* mice fed with normal diet (ND) or Western diet (WD) after treatment with acetone or 7,12-dimethylbenz[a]anthracene (DMBA). (**E-F**) Relative ratio sum in serum of **(E)** free fatty acids (FFA) and **(F)** lysophosphatidylcholine (LPC) of WT and *Plin5^-/-^* mice fed with ND or a WD after treatment with acetone or DMBA. Data are depicted as Box-Whisker plots representing the smallest and largest values (n=3-8). The line represents the median. Statistical significance was determined using the Kruskal-Wallis test. * *p* <0.05, ** *p* <0.01, *** *p* <0.001.

**
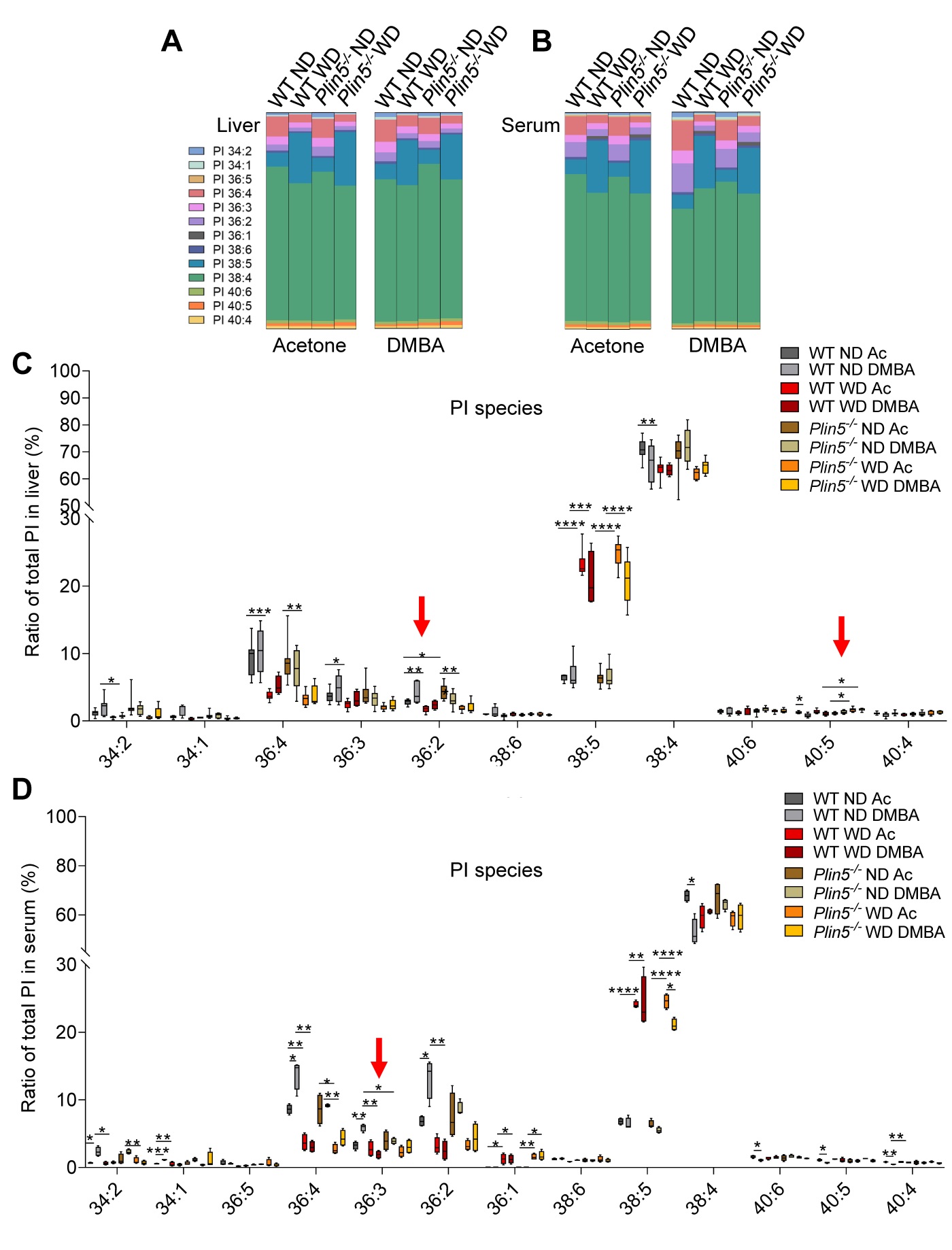
**

**Suppl. Figure 4: The phosphatidylinositol profiles are not different in wild-type and *Plin5^-/-^* mice.** Visual representation of phosphatidylinositol (PI) species in **(A)** liver and **(B)** serum samples of WT and *Plin5^-/-^* mice fed a normal (ND) or Western diet (WD) after treatment with DMBA or acetone (Ac). **(C)** Ratio of total PI in the liver represented as percentage (n=4 biological samples). **(D)** Ratio of total PI in serum (n=4 biological samples). Red arrows indicate differences found between WT and *Plin5^-/-^* mice. For figures **(C-D)**, data is depicted as Box-Whisker-plots representing the smallest and largest values. The line represents the median. Statistical significance was determined using the Holm-Sidak method with α=0.05. * *p* <0.05, ** *p* <0.01, *** *p* <0.001, **** *p* <0.0001.


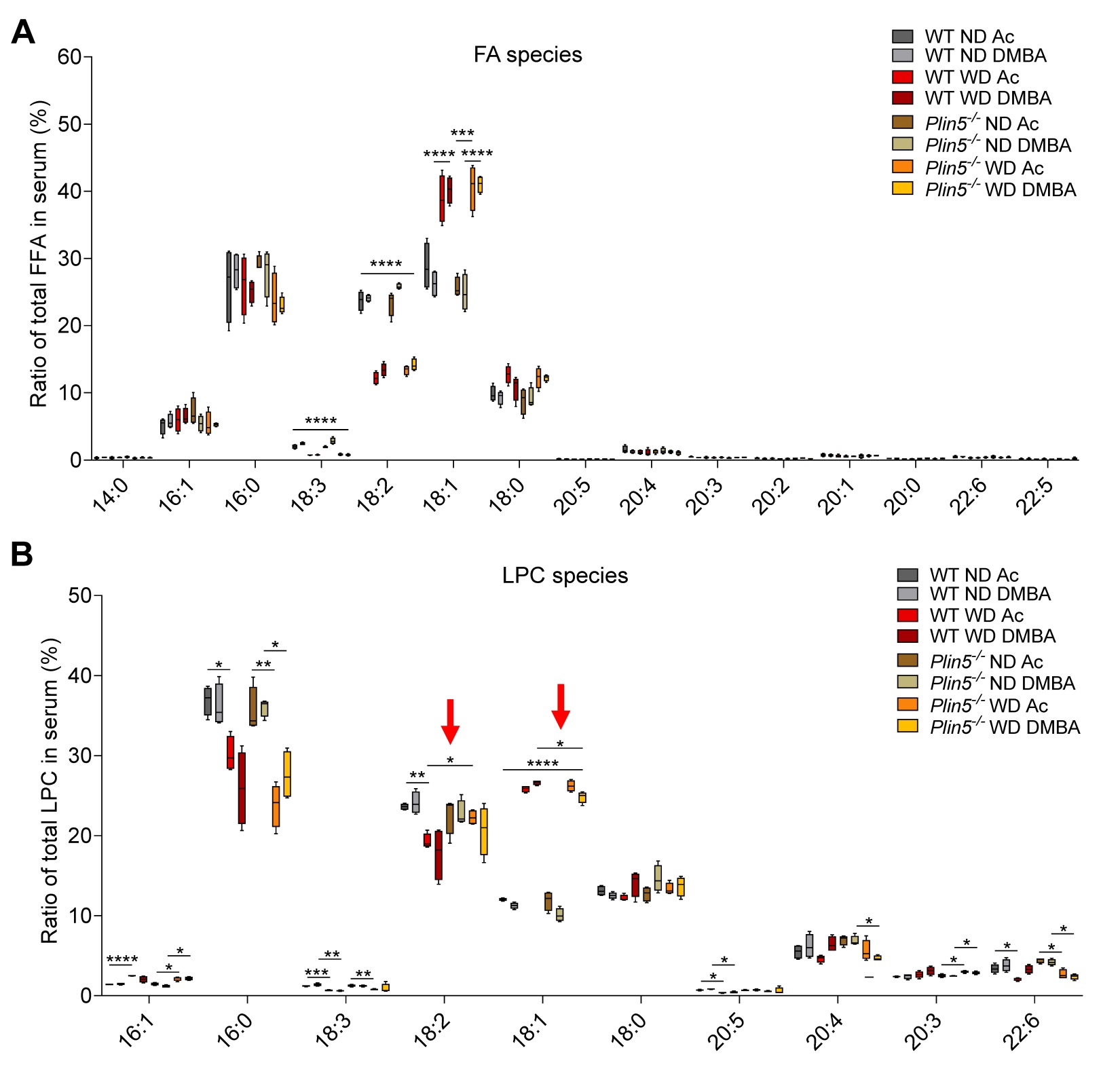


**Suppl. Figure 5: Free fatty acids in serum and lysophosphatidylcholines ratios are not altered after the loss of *Plin5*.** Ratio of total **(A)** free fatty acids (FFA) and **(B)** total lysophosphatidylcholines (LPC) in serum represented as percentage (n=4 biological samples). Mice were fed a control (ND) or a Western diet (WD) after treatment with DMBA or acetone (Ac). Red arrows indicate differences between WT and *Plin5^-/-^* mice. Data are depicted as Box-Whisker-plots with whiskers representing the smallest and largest values. The lines represent the median. Statistical significance was determined using the Holm-Sidak method with α=0.05. * *p* <0.05, ** *p* <0.01, *** *p* <0.001, **** *p*<0.0001.


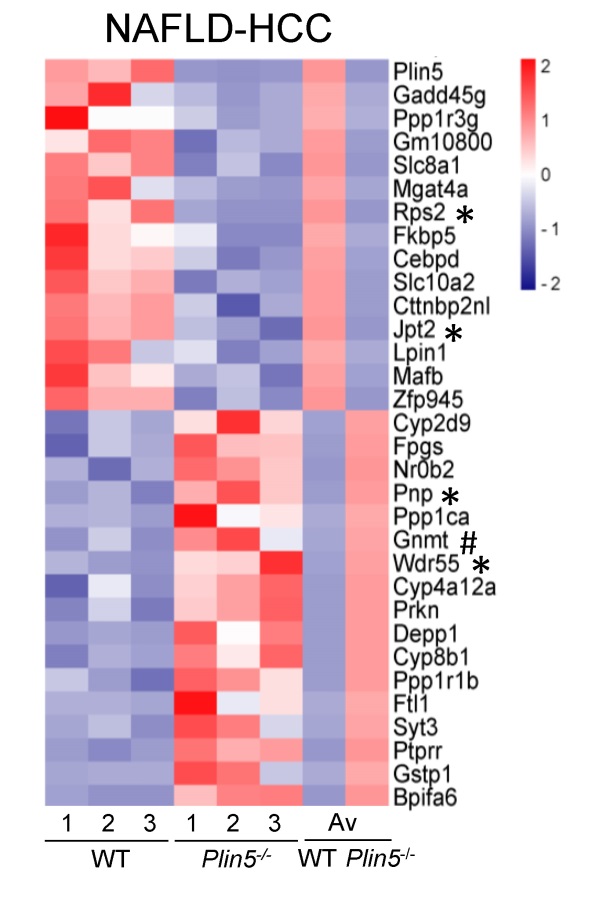


**Suppl. Figure 6: The loss of *Plin5* does not drive transcriptional alterations in the NAFLD-HCC model.** Heat map of differentially expressed genes altered during NAFLD-HCC. * genes considered an unfavorable prognosis in HCC, # genes considered a favorable prognosis according to the Human Protein Atlas.


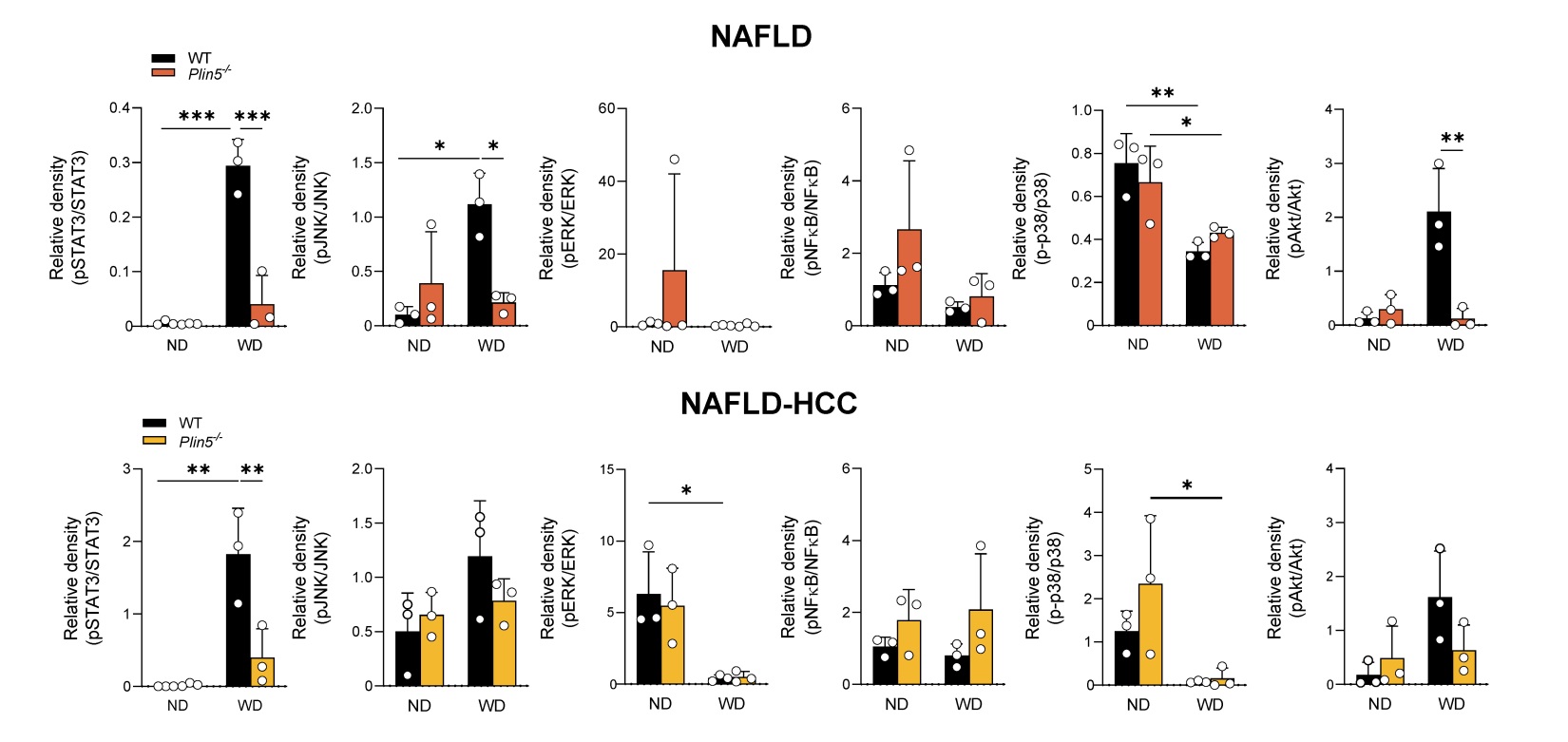


**Suppl. Figure 7: Statistics to Western blot analysis depicted in Figure 6.** The Western blot signals from Figure 6 were determined densitometrically using ImageJ software. The ratio of phosphorylated to total protein was determined for each protein. * *p* <0.05, ** *p* <0.01, *** *p* <0.001.
