# Supplementary figures and images for: Perilipin 5 deletion protects against nonalcoholic fatty liver disease and hepatocellular carcinoma by modulating lipid metabolism and inflammatory responses
Source: Cell Death Discov. 2024 Feb 22;10:94. doi: 10.1038/s41420-024-01860-4 (PMC10884415; doi:10.1038/s41420-024-01860-4)

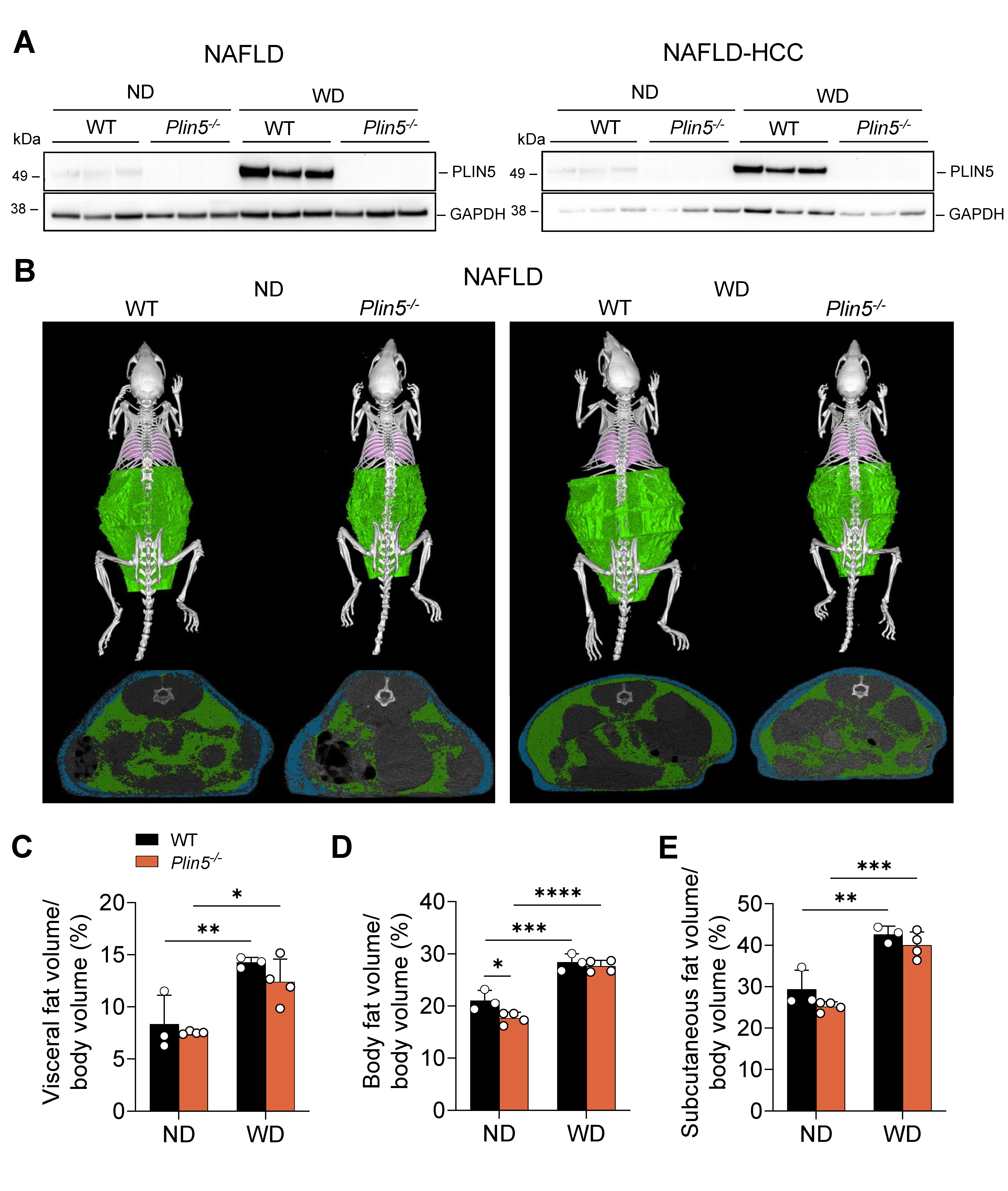

Supplement: Supplementary file 2 — Supplementary Figure 1 [file 41420_2024_1860_MOESM2_ESM.jpg]

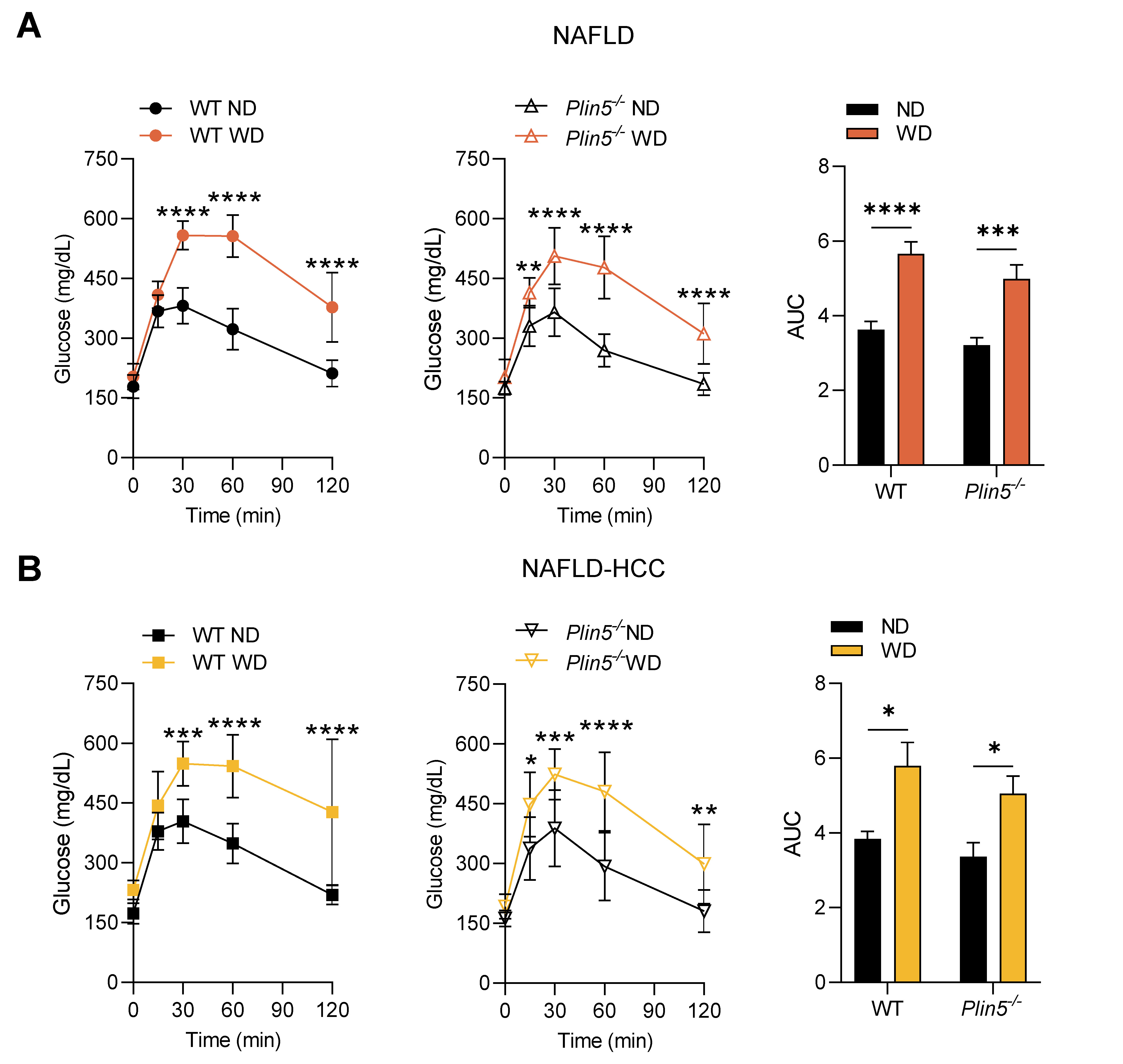

Supplement: Supplementary file 3 — Supplementary Figure 2 [file 41420_2024_1860_MOESM3_ESM.jpg]

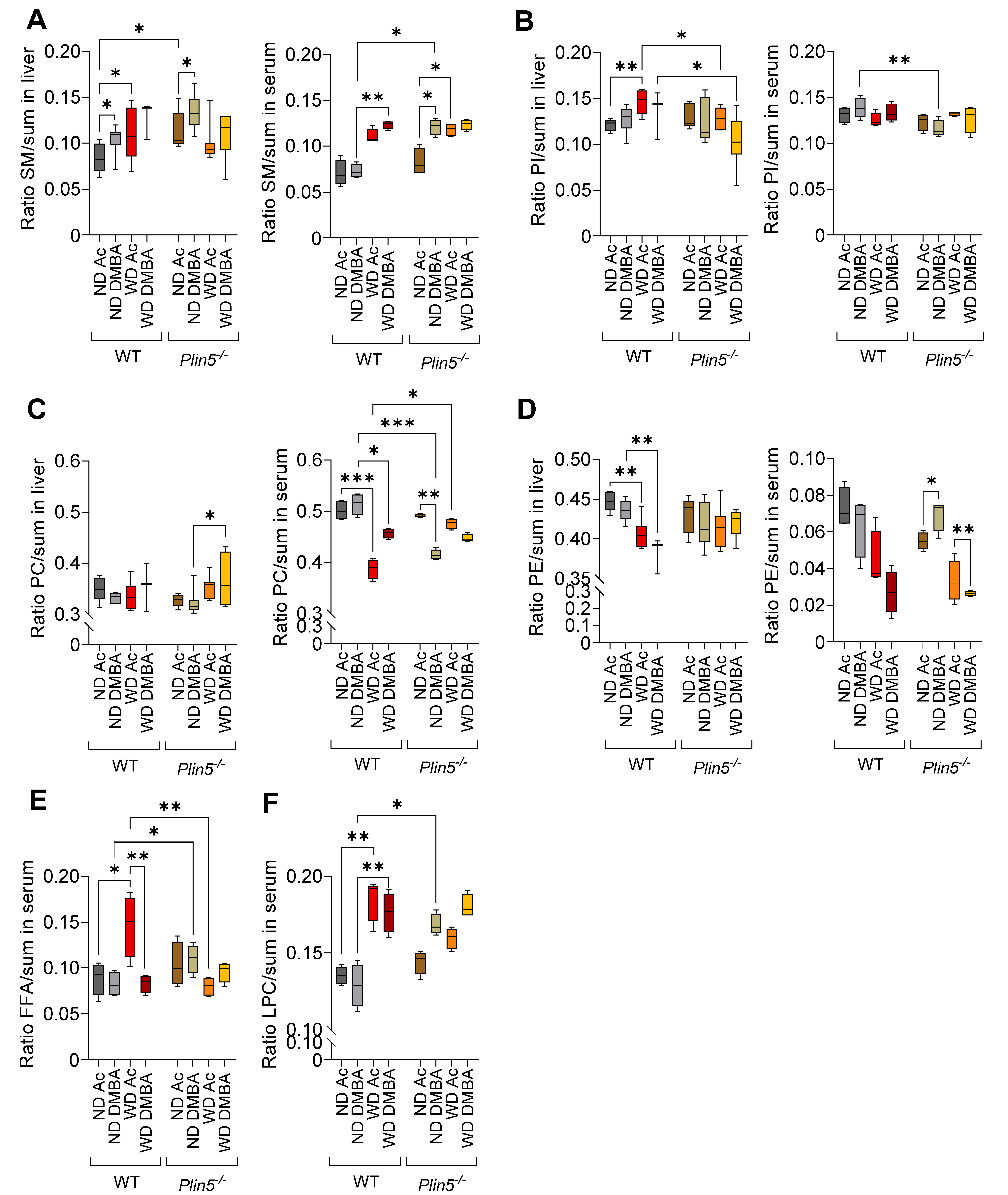

Supplement: Supplementary file 4 — Supplementary Figure 3 [file 41420_2024_1860_MOESM4_ESM.jpg]

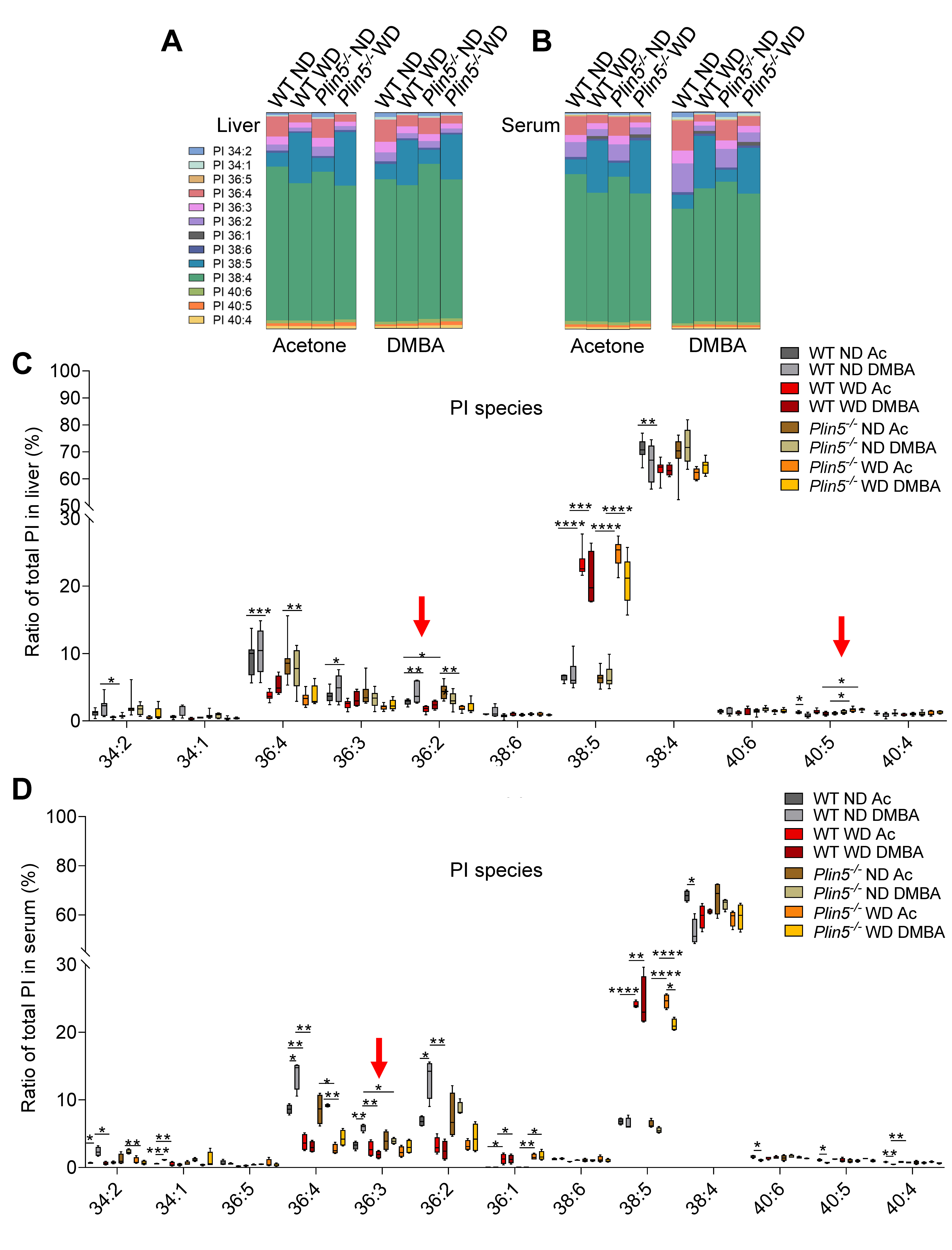

Supplement: Supplementary file 5 — Supplementary Figure 4 [file 41420_2024_1860_MOESM5_ESM.jpg]

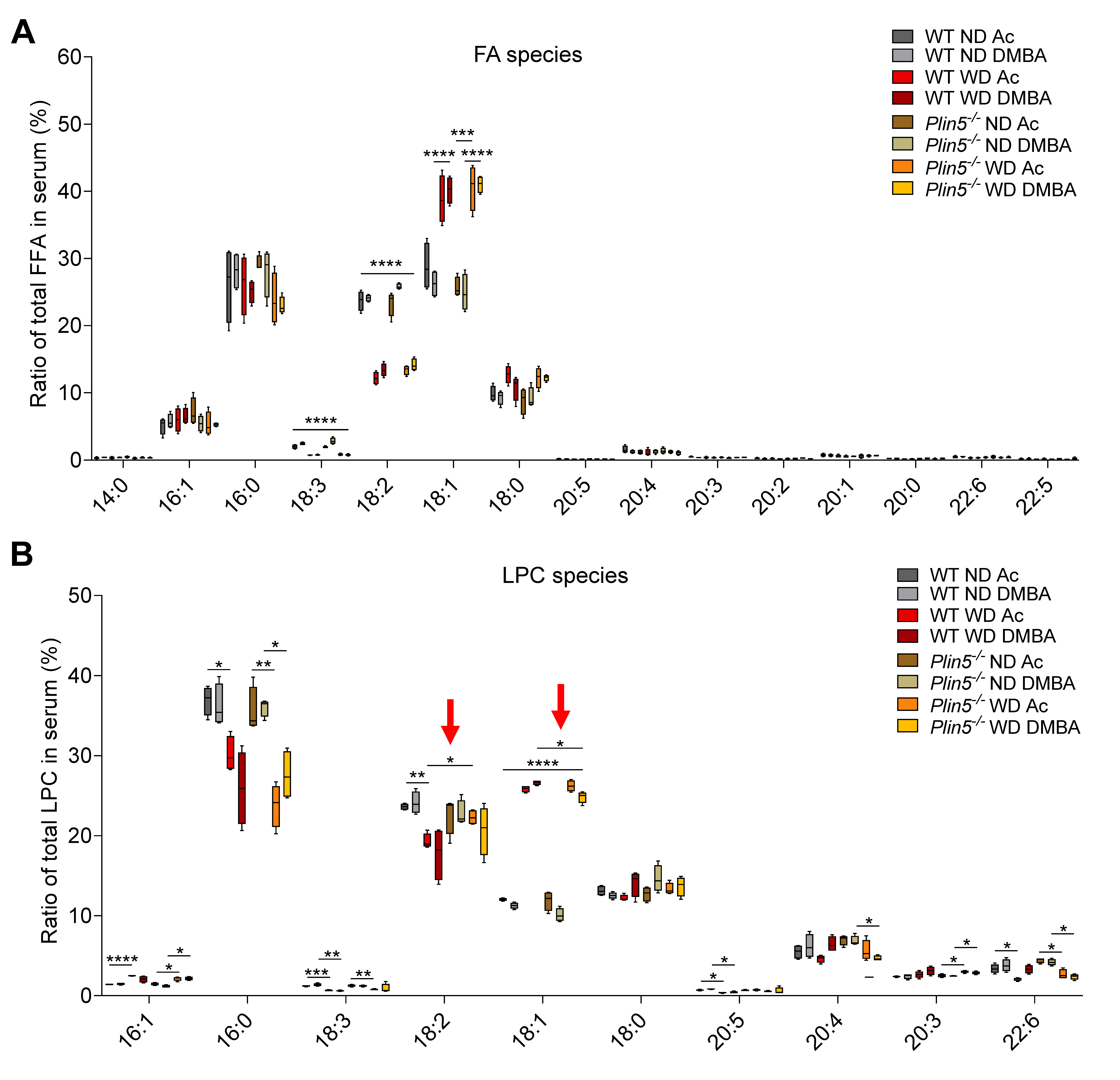

Supplement: Supplementary file 6 — Supplementary Figure 5 [file 41420_2024_1860_MOESM6_ESM.jpg]

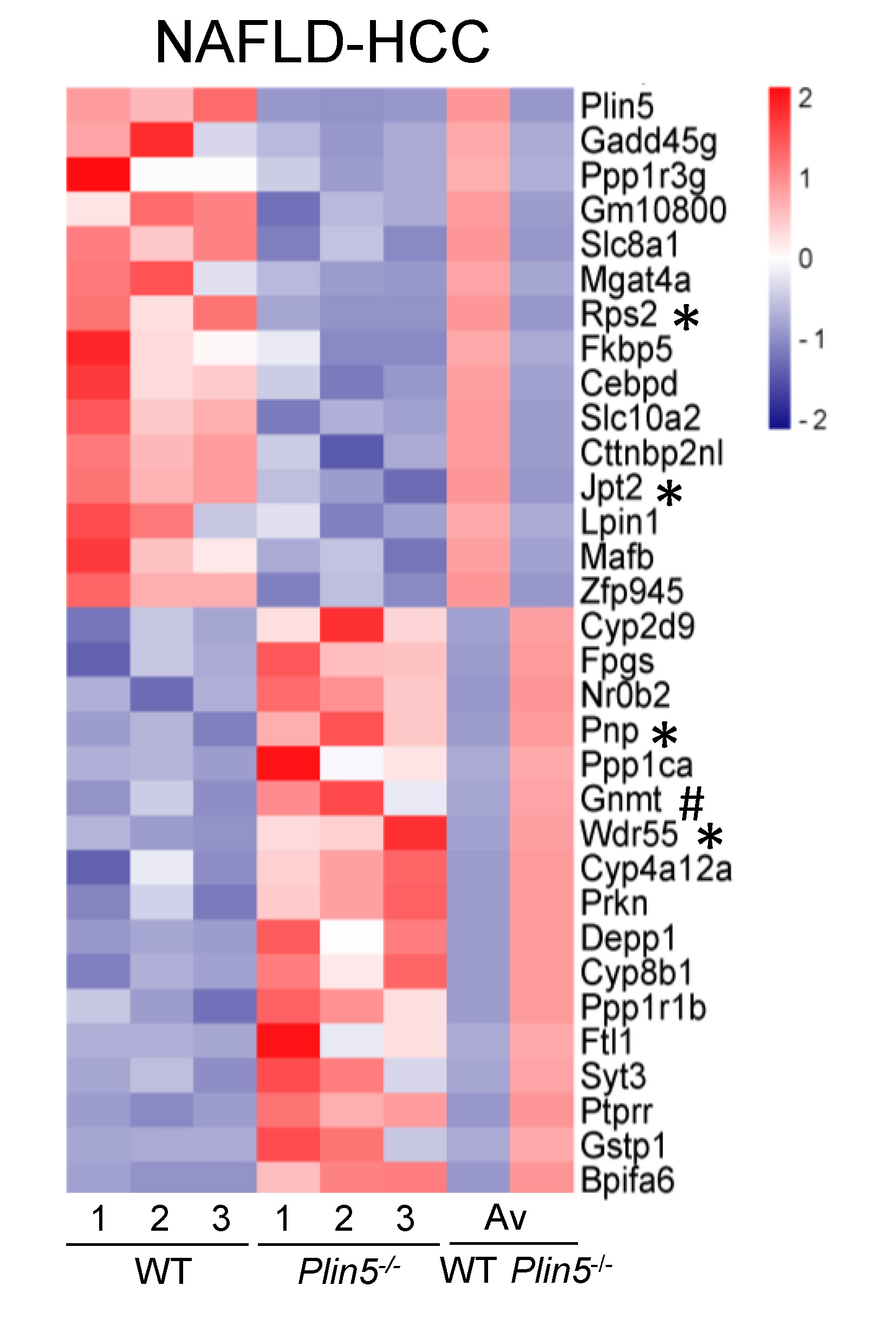

Supplement: Supplementary file 7 — Supplementary Figure 6 [file 41420_2024_1860_MOESM7_ESM.jpg]

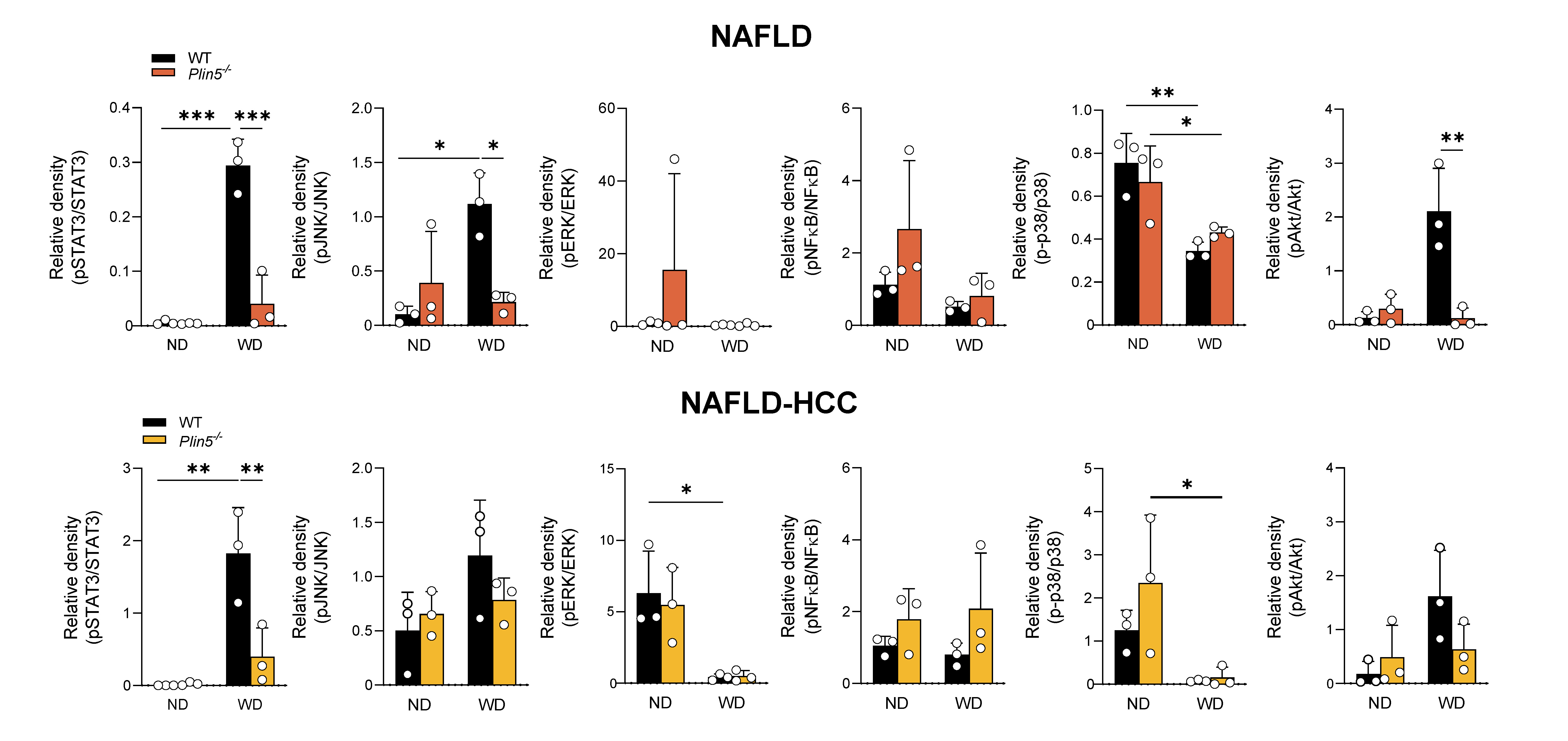

Supplement: Supplementary file 8 — Supplementary Figure 7 [file 41420_2024_1860_MOESM8_ESM.jpg]
