# Supplementary material for: Perilipin 5 deletion protects against nonalcoholic fatty liver disease and hepatocellular carcinoma by modulating lipid metabolism and inflammatory responses
Source: Cell Death Discov. 2024 Feb 22;10:94. doi: 10.1038/s41420-024-01860-4 (PMC10884415; doi:10.1038/s41420-024-01860-4)

**Perilipin 5 deletion protects against nonalcoholic fatty liver disease (NAFLD) and NAFLD-induced hepatocellular carcinoma through modulation of lipid metabolism and inflammatory response in murine models**

Paola Berenice Mass-Sanchez^1¶^, Marinela Krizanac^1¶^, Paula Štancl^2^, Marvin Leopold^3^, Kathrin Engel^3^, Eva Miriam Buhl^4^, Josef van Helden^5^, Nikolaus Gassler^6^, Jürgen Schiller^3^, Rosa Karlić^2^, Diana Möckel^7^, Twan Lammers^7^, Steffen K. Meurer^1^, Ralf Weiskirchen^1^*, and Anastasia Asimakopoulos^1^*

^1^ Institute of Molecular Pathobiochemistry, Experimental Gene Therapy and Clinical Chemistry (IFMPEGKC), RWTH University Hospital Aachen, D-52074 Aachen, Germany

^2^ Division of Molecular Biology, Department of Biology, Faculty of Science, University of Zagreb, HR-10000, Zagreb, Croatia

^3^ Institute for Medical Physics and Biophysics, Leipzig University, Faculty of Medicine, D-04107 Leipzig, Germany

^4^ Electron Microscopy Facility, Institute of Pathology, RWTH Aachen University Hospital, D-52074 Aachen, Germany

^5^ Laboratory Mönchengladbach-MVZ Dr. Stein and Colleagues, D-41169 Mönchengladbach, Germany

^6^ Section Pathology, Institute of Legal Medicine, University Hospital Jena, D-07747 Jena, Germany

^7^ Institute for Experimental Molecular Imaging, RWTH Aachen, D-52074 Aachen, Germany

To whom correspondence should be addressed: [rweiskirchen@ukaachen.de](mailto:rweiskirchen@ukaachen.de) or [aasimakopoulou@ukaachen.de](mailto:aasimakopoulou@ukaachen.de)

**Supplementary Figures**

- Original Western blots Figure 3I
- Original Western blots Figure 6A and Figure 6B
- Original Western Blots Figure S1A

***Notes:***

***Left:* transmission light; *right:* chemiluminescence images.**

When multiple samples were run on the same membrane, out of which some were not used for the Figures in the manuscript, the membranes were cropped to depict only the part of the membrane used for the Figures in the manuscript.

When possible, one membrane section was incubated successively with different antibodies.

When multiple membranes were run, GAPDH was detected for all membranes, but only one GAPDH was taken as a „representative” for the manuscript.

**Original Western blots Figure 3I**


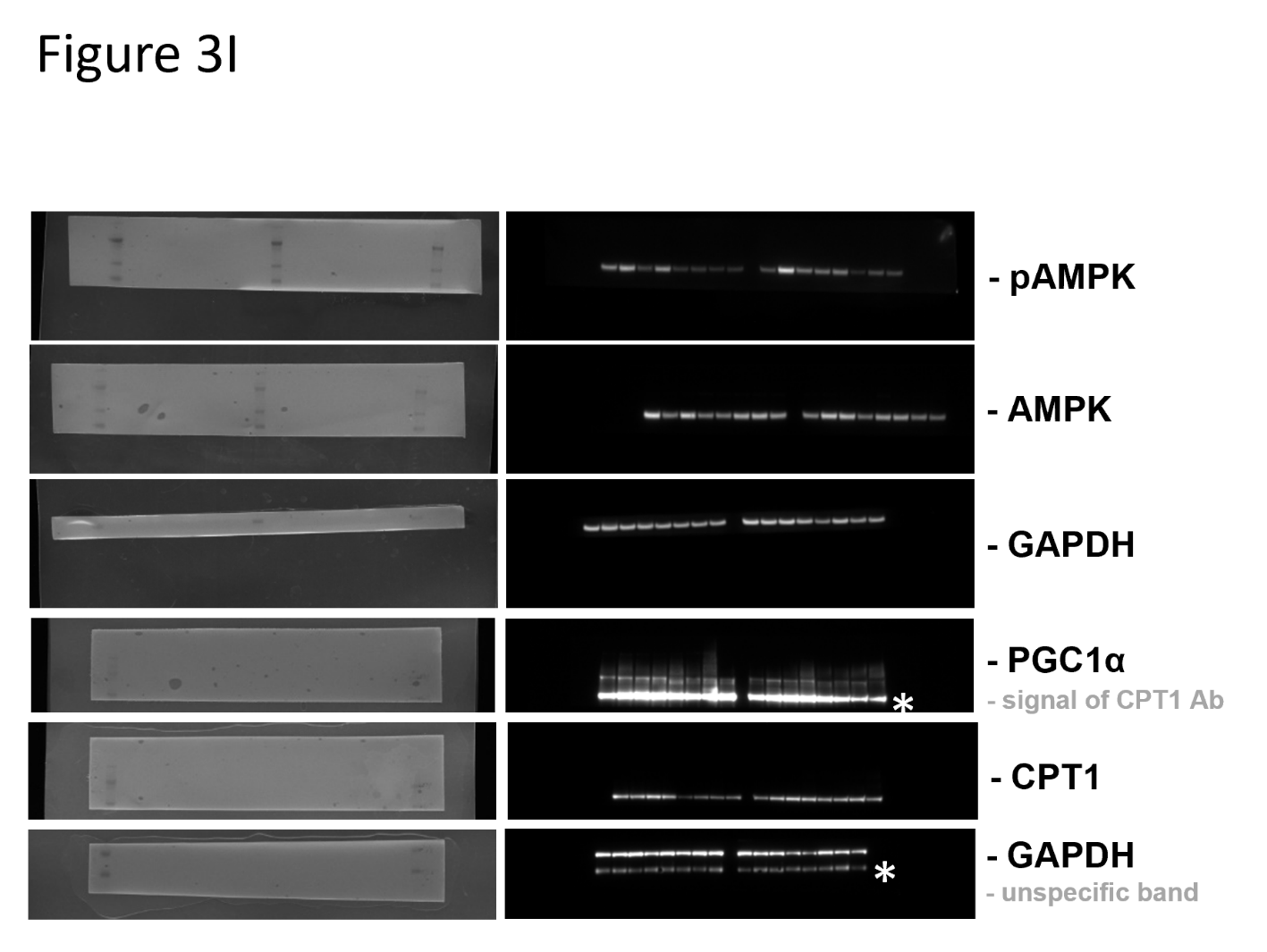


**Original Western blots Figure 6A and Figure 6B**


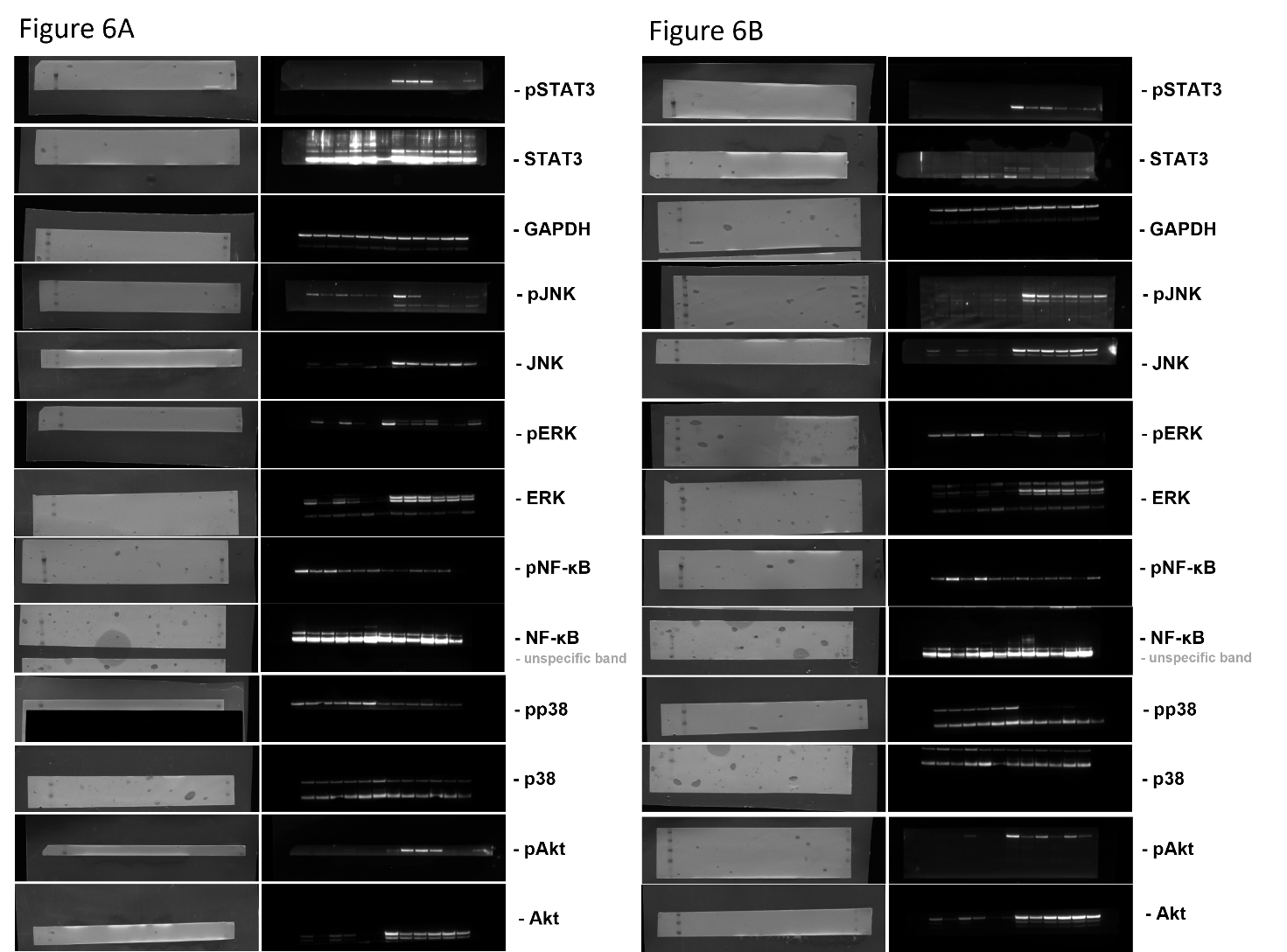


**Original Western blots Figure S1A**


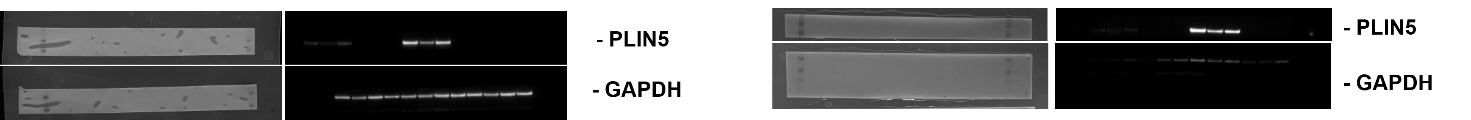

Supplement: Supplementary file 9 — Original Data File [file 41420_2024_1860_MOESM9_ESM.docx]
